# A novel batch effect correction framework for robust integration of high-variance data via a global-information virtual reference batch

**DOI:** 10.3389/fmicb.2026.1877381

**Published:** 2026-08-12

**Authors:** Yuqian Liu, Lan Du, Jingxuan Jiang, Xin Tong, Junfei Xu, Jiayin Wang, Xin Lai

**Affiliations:** 1 School of Computer Science and Technology, Faculty of Electronics and Information Engineering, Xi’an Jiaotong University, Xi’an, Shaanxi, China; 2 Shaanxi Engineering Research Center of Medical and Health Big Data, Xi’an Jiaotong University, Xi’an, Shaanxi, China; 3 School of Software Engineering, Faculty of Electronics and Information Engineering, Xi’an Jiaotong University, Xi’an, Shaanxi, China

**Keywords:** batch effect correction, ComBat, linear model, nearest neighbor pair, interaction-rich biological systems

## Abstract

The integration of multi-batch high-variance datasets is increasingly important in studies of complex biological systems. In application domains such as microbial symbiosis, host–microbe interactions, and ecosystem robustness, this places a stringent demand on batch correction methods which must reduce technical batch effects while preserving the biologically meaningful cross-sample structure required for downstream interpretation. Here, we present GIR-Combat, a novel batch correction framework that constructs a global-information virtual reference batch from shared cross-batch structure, thereby enabling more consistent and objective correction across datasets. GIR-Combat identifies mutually nearest neighbors across batches, leverages their shared information to define a virtual reference, and incorporates this reference into a linear modeling framework for correction. By transforming reference-batch specification from a subjective choice into a modeling step, GIR-Combat provides a more objective and robust solution for correcting high-variance and compositionally imbalanced datasets in which conventional methods frequently underperform. We evaluated GIR-Combat on simulated datasets and multiple public benchmark datasets. The results show that GIR-Combat improves batch correction performance relative to existing methods, achieving better batch correction while preserving biologically meaningful structure. Quantitative and visual evaluation metrics further demonstrate its robustness and scalability in challenging integration scenarios. Overall, GIR-Combat provides a practical and methodologically grounded framework for high-variance multi-batch data integration, with potential value in applications where reliable integrated representations are required for interpreting complex biological interactions.

## 1 Introduction

High-throughput omics technologies have advanced the study of complex biological systems by enabling increasingly fine-grained molecular profiling across diverse scenarios. As datasets accumulate across laboratories, platforms, and time points, multi-batch integration has become indispensable for improving reproducibility and enabling biologically meaningful comparisons across studies (Lazar et al., 2013; Yu et al., 2023; Čuklina et al., 2021). This challenge is particularly acute in applications characterized by high heterogeneity and complex interaction structures, including host-associated and environmental systems, where downstream inference often depends on the ability to distinguish structured biological variation from technical batch effects (Egan et al., 2020; Franzosa et al., 2015; Kobel et al., 2024; Chetty and Blekhman, 2024). Accordingly, batch correction is required not only to reduce technical variation, but also to preserve the cross-sample structure on which reliable biological interpretation depends.

Batch effects remain especially difficult to resolve. Systematic variation introduced during sample processing, library preparation, sequencing, and platform-specific workflows can distort sample relationships, confound biological interpretation, and generate spurious signals in downstream analyses if left uncorrected (Lazar et al., 2013; Yu et al., 2023; Li et al., 2020). These effects are further amplified in high-variance datasets and in compositionally imbalanced settings, where uneven representation of biological groups across batches complicates the separation of technical and biological sources of variation (Yu et al., 2024; Phua et al., 2022). Hence, the demands placed on correction methods become more stringent. They must mitigate batch-associated distortion without erasing the biologically meaningful structure required for downstream analysis.

A broad range of correction methods has been developed to address batch effects. ComBat is one of the most widely used methods for batch effect correction due to its computational efficiency, theoretical robustness, and practical ease of use (Adamer et al., 2022a; Johnson et al., 2007). ComBat combines a linear modeling framework with empirical Bayes estimation to adjust for batch-specific mean shifts, and has been successfully applied to proteomic, cross-sectional and cross-platform genomic studies (Čuklina et al., 2021; Walker et al., 2008; Chmielewski et al., 2014). In particular, it has proven effective for integrating data generated from different microarray platforms, such as Illumina and Affymetrix microarray data (Turnbull et al., 2012). ComBat has also been reported to outperform several alternative correction strategies, including Distance-weighted Discrimination (DWD) (Benito et al., 2004), Mean-centering approaches (such as PAMR) (Sims et al., 2008), Surrogate Variable Analysis (SVA) (Jaffe et al., 2015; Leek and Storey, 2007), and ratio-based normalization methods (Chen et al., 2011; Luo et al., 2010), especially when batch sizes are small or comparable.

Despite these strengths, conventional ComBat has limitations, particularly in its reliance on selecting a reference batch, which can introduce subjectivity into the correction process. The challenge is further compounded when dealing with datasets where an optimal reference batch is unavailable, leading to suboptimal correction outcomes. In Zhang et al. (2018) study, the author identified certain challenges associated with the traditional ComBat approach. One such challenge is that ComBat removes batch effects that impact both the means and variances of each gene across the batches, however, there are instances where the data might require a less (or more) extreme batch adjustment. To address this issue, Zhang et al. (2018) proposed the incorporation of a reference batch within the ComBat model and expounded on its importance in relation to batch effect correction. The authors underscored the crucial role of the reference batch, particularly in the context of biomarker studies. For instance, a single dataset is used for training a fixed biomarker, which is subsequently applied across multiple disparate batches or datasets, potentially even at different time points. The method proposed by Zhang et al. (2018) effectively mitigates the detrimental effects of test set bias during the generation of biomarker signatures. However, in the majority of prevailing batch effect correction methods, the selection of reference batches typically involves choosing a single batch from the entire dataset to serve as the reference batch. Although this method allows for a benchmark in the calibration process, the selection of such a benchmark is highly subjective. Moreover, in certain scenarios, relying solely on a single batch as a reference may prove insufficient in significantly enhancing the efficacy of batch adjustment. Wynn L. Walker et al. emphasized that an experimental design would ideally include more than one reference sample per batch as a preventative measure in case one of the reference samples was of poor quality (Li et al., 2020). In batch effect correction, each batch contains useful information about the underlying biological signal, such as gene expression patterns or cellular subpopulations, which are relevant to the research question at hand. Hicks, S. C. et al. underscored the significance of preserving the inherent biological signal within each batch while mitigating technical biases during batch effect correction (Hicks and Irizarry, 2015). When dealing with batches containing less information, it is essential to ensure that the batch effect correction method is robust and can handle different types of information across batches.

More recent developments in single-cell batch correction have sought to address related limitations, but important challenges remain (Hicks et al., 2018; Tran et al., 2020; Korsunsky et al., 2019; Haghverdi et al., 2018). For example, Harmony (Korsunsky et al., 2019) employs clustering and optimization techniques to adjust latent spaces, balancing batch effects with biological variation. While effective in some cases, this method may lack consistency across different datasets, limiting its generalizability. Similarly, Mutual Nearest Neighbors (MNN) (Haghverdi et al., 2018) identifies nearest neighbor pairs across batches to correct local batch effects, but it struggles with global shifts and is sensitive to noise and sample size imbalances between batches. Taken together, these limitations indicate that existing methods still leave unresolved the problem of how to perform robust batch correction when high variance, compositional imbalance, and the lack of a clearly representative reference batch coexist.

These limitations are especially consequential in research areas such as microbial symbiosis, host–microbe interactions, and ecosystem robustness, where reliable downstream interpretation depends on integrated data that retain biologically meaningful cross-sample structure (Franzosa et al., 2015; Kobel et al., 2024; Chetty and Blekhman, 2024). From this perspective, advances in reference-based correction are important not only for batch adjustment itself, but also for ensuring that downstream analyses are built on a more reliable integrated representation of the data.

These considerations motivate the need for a framework that can exploit the biologically meaningful information distributed across batches, rather than treating reference specification as a fixed external choice. To address the subjectivity associated with manually specifying reference batches and the challenges posed by large variances, we present GIR-Combat (Global Information Reference-Combat), a novel batch effect correction method designed to overcome the limitations of existing approaches. GIR-Combat introduces a global-information virtual reference batch from shared cross-batch structure. Specifically, the framework identifies mutually nearest neighbors across batches, uses their shared information to define a biologically informed virtual reference, and incorporates that reference into a linear modeling framework for correction. The contribution of GIR-Combat lies not in the use of a virtual batch, but in transforming reference specification from an externally imposed choice into an inferential step guided by shared cross-batch biological correspondence. The resulting virtual reference batch is a data-driven reference representation derived from MNN-supported shared cross-batch structure and used as the calibration target for subsequent model-based correction. This distinction is critical in high-variance and compositionally imbalanced datasets, where the reference itself is often the least stable component of reference-based correction. By aligning samples with comparable biological attributes across batches, GIR-Combat preserves biologically meaningful structure while reducing the risk of erroneous cross-batch associations. The framework is not only applicable to a single data modality but also sample-by-feature data matrices in which biologically comparable groups are shared across batches or can be approximated through clustering. We evaluate GIR-Combat on both simulated and real datasets, showing its improved effectiveness and practicality. It offers an adaptable and scalable solution for high-variance omics integration where reliable cross-batch comparability is essential for downstream biological interpretation.

## 2 Methods

### 2.1 The conventional reference batch correction model

Zhang et al. (2018) proposed a reference-based batch correction approach that uses one batch as the baseline for the batch adjustment. This formulation is particularly useful when one batch is intended to serve as a fixed reference set. However, because the reference batch must be specified in advance, the method remains dependent on an externally imposed choice.

Suppose the data contain m batches, within batch i containing $n_{i}$ samples for i=1,…,m , and let g=1,…,G index measured sample attributes. The model is specified in Equation 1.

$$\;Y_{\mathrm{ijg}}=\alpha_{\mathrm{rg}}+X_{\mathrm{ij}}\beta_{\mathrm{rg}}+\gamma_{\mathrm{rig}}+\delta_{\mathrm{rig}}\varepsilon_{\mathrm{ijg}}\;\;\;\text{where}\;\;\varepsilon_{\mathrm{ijg}}∼N(0,\sigma_{\mathrm{rg}}^{2})$$ (1)

Where the raw data $Y_{\mathrm{ijg}}$ is the value of $g^{\mathrm{th}}$ attribute in the $j^{\mathrm{th}}$ sample of the $i^{\mathrm{th}}$ batch. In transcriptomic applications, these attributes correspond to genes. More generally, they may represent molecular attributes in a sample-by-feature data matrix.

Assume that the reference batch is artificially set to the $r^{\mathrm{th}}$ batch. $\alpha_{\mathrm{rg}}$ represents the average value of the $g^{\mathrm{th}}$ attribute in the chosen reference batch ( r ). The matrix $X_{\mathrm{ij}}$ are the sample condition design matrix of regression coefficients $\beta_{\mathrm{rg}}$ . The tensor $\varepsilon_{\mathrm{ijg}}$ denotes the error term for the linear model fit, with a normal distribution assumption of $\varepsilon_{\mathrm{ijg}}$ following $\varepsilon_{\mathrm{ijg}}∼N(0,\sigma_{\mathrm{rg}}^{2})$ . $\gamma_{\mathrm{rig}}$ and $\delta_{\mathrm{rig}}$ represents the additive and multiplicative batch differences between the reference batch r and batch i for attribute g .

Begin by standardizing the data on an attribute-wise to ensure attributes display comparable overall means and variances. Proceed to determine the model parameters $\alpha_{\mathrm{rg}}$ , $\beta_{\mathrm{rg}}$ , and $\gamma_{\mathrm{rig}}$ , denoted as ${\hat{\alpha }}_{\mathrm{rg}}$ , ${\hat{\beta }}_{\mathrm{rg}}$ , and ${\hat{\gamma }}_{\mathrm{rig}}$ for i=1,…,m and g=1,…,G . Apply an attribute-wise ordinary least-squares method for this purpose, with the constraint g=1,…,G (applicable to all g=1,…,G ) to guarantee the identifiability of these parameters. Subsequently, compute ${\hat{\sigma }}_{\mathrm{rg}}^{2}$ as ${\hat{\sigma }}_{\mathrm{rg}}^{2}=\frac{1}{N}\sum_{\mathrm{ij}}{\left(Y_{\mathrm{ijg}}-{\hat{\alpha }}_{\mathrm{rg}}-X_{\mathrm{ij}}{\hat{\beta }}_{\mathrm{rg}}-{\hat{\gamma }}_{\mathrm{rig}}\right)}^{2}$ ( N is the total number of samples). The standardized data $Z_{\mathrm{ijg}}$ , are now calculated by

$$Z_{\mathrm{ijg}}=\frac{Y_{\mathrm{ijg}}-{\hat{\alpha }}_{\mathrm{rg}}-X_{\mathrm{ij}}{\hat{\beta }}_{\mathrm{rg}}}{{\hat{\sigma }}_{\mathrm{rg}}}$$ (2)

Assume that the standardized data, $Z_{\mathrm{ijg}}$ , satisfy the distributional form, $Z_{\mathrm{ijg}}∼N(\omega_{\mathrm{rig}},\delta_{\mathrm{rig}}^{2})$ . assume the parametric forms for prior distributions on the batch effect parameters to be $\omega_{\mathrm{rig}}∼N(Y_{i},\tau_{i}^{2})$ and $\delta_{\mathrm{rig}}^{2}∼\text{Inverse Gamma}(\lambda_{i},\theta_{i})$ . The hyperparameters $Y_{i}$ , $\tau_{i}^{2}$ , $\lambda_{i}$ , $\theta_{i}$ are estimated empirically from standardized data using the method of moments. Based on the above distribution assumption, the Empirical Bayes (EB) estimates of batch effect parameters $\omega_{\mathrm{rig}}$ and $\delta_{\mathrm{rig}}^{2}$ are given by the conditional posterior mean, respectively. These EB estimates provide a shrinkage-based adjustment of the additive and multiplicative batch effects, allowing more stable inference in small-sample scenarios, as shown in Equations 3, 4.

$$\omega_{\mathrm{rig}}^{*}=\frac{n_{i}{\bar{\tau }}_{i}^{2}{\hat{\omega }}_{\mathrm{rig}}+\delta_{\mathrm{rig}}^{2*}{\bar{Y}}_{i}}{n_{i}{\bar{\tau }}_{i}^{2}+\delta_{\mathrm{rig}}^{2*}}$$ (3)

$$\delta_{rig}^{2*}=\frac{{\bar{\theta }}_{i}+\frac{1}{2}\underset{j}{\sum }\;{\left(Z_{ijg}-\omega_{rig}^{*}\right)}^{2}}{\frac{n_{j}}{2}+{\bar{\lambda }}_{i}-1}$$ (4)

After calculating the adjusted batch effect estimators, $\omega_{\mathrm{rig}}^{*}$ and $\delta_{\mathrm{ig}}^{2*}$ , now adjust the data. The EB batch-adjusted data $Y_{\mathrm{ijg}}^{*}$ can be calculated using EB estimated batch effects by Equation 5.

$$Y_{\mathrm{ijg}}^{*}=\frac{{\hat{\sigma }}_{\mathrm{rg}}}{{\hat{\delta }}_{\mathrm{rig}}^{*}}(Z_{\mathrm{ijg}}-{\hat{\omega }}_{\mathrm{rig}}^{*})+{\hat{\alpha }}_{\mathrm{rg}}+X_{\mathrm{ij}}{\hat{\beta }}_{\mathrm{rg}}$$ (5)

Following the batch effect adjustment, the reference batch remains unchanged, while the means and variances of the non-reference batches will be adjusted to the mean/variance of the reference batch. Designating one of the batches as a reference batch from all batches proves advantageous in situations where one batch is larger or of better quality. In addition, this will be useful in biomarker situations where the researcher wants to fix the training set/model and then adjust test sets to the reference/training batch. This avoids test set bias in such studies (Leek et al., 2012). This also allows batches of data to be adjusted at different times without impacting the results. This approach will be advantageous to data generating consortiums where data arrive sequentially in small batches. The main limitation of this formulation is that the reference batch is not inferred from the data, but specified externally. It relies on researchers to designate the reference batch, largely depends on their expertise in handling the issue and introduces a degree of subjectivity. Correction performance depends on the representativeness of the chosen batch, which may be problematic in high-variance and compositionally imbalanced datasets. Furthermore, it overlooks the biological information contained in other batches.

### 2.2 GIR-combat model

GIR-Combat is designed to address the instability introduced by manually specified reference batches in high-variance and compositionally imbalanced datasets. The framework is formulated for general sample-by-feature data matrices, where rows or columns represent samples depending on the data layout, and features correspond to measured molecular attributes. Single-cell transcriptomic data constitute one important application setting, but the method is not restricted to that modality. The methodological contribution of GIR-Combat lies in replacing externally imposed reference-batch selection with a data-driven virtual reference constructed from shared cross-batch structure. In this formulation, the virtual reference serves as a calibration target derived from MNN-supported cross-batch correspondence.

#### 2.2.1 Construction of the global information reference batch

To construct the global-information virtual reference batch, GIR-Combat first partitions the input matrix into sub-matrices corresponding to each experimental batch. To align the samples across batches, we first apply cosine normalization to each submatrix to ensure that the expression vectors are scaled to the unit length. This standardization promotes the identification of biologically comparable samples across batches by reducing technical differences in magnitude. Next, the mutual nearest neighbor (MNN) pairs between the first two batches are identified based on the cosine similarity between the normalized expression vectors. These MNN pairs are used as anchor points to estimate batch-specific correction vectors, and then are used to coordinate the first two batches. The corrected batches are then integrated into the virtual reference batches to provide a unified framework for the iterative calibration of the remaining batches.

Consider the first two batches, denoted batch 1 and batch 2. Taking Batch 1 as the temporary benchmark, Batch 2 shall be calibrated with reference to Batch 1. The difference between the paired samples is attributed to the batch effect, and the difference in expression values between the nearest neighbor pairs of samples provides an estimate of the batch effect. After traversing all samples within the first two batches, a sample pair list is generated to calculate the batch effect correction vector ${\vec{v}}^{\mathrm{ml}}$ . Assuming a mutually nearest neighbor pair, where m originates from batch 1 and l from batch 2. The feature profiles ${\vec{E}}^{m}$ and ${\vec{E}}^{l}$ represent the values of all attributes for samples m and l , respectively. The batch effect correction vector for such a mutually nearest neighbor pair is outlined in Equation 6 (Haghverdi et al., 2018).

$${\vec{v}}^{\mathrm{ml}}={\vec{E}}^{m}-{\vec{E}}^{l}$$ (6)

It is worth noting that in batch-effect correction, MNN alone can effectively deal with local matching problems, but it has some significant shortcomings. First, MNN has limited processing power for global systematic migration and may not be able to eliminate mean and variance differences between batches. In addition, MNN is more sensitive to noise and data sparsity, which may lead to inaccurate pairing, thus affecting the correction effect (Nguyen et al., 2023). In addition, MNN assumes a uniform distribution of samples between batches, which may not be true in practice, thus limiting its applicability (Luecken et al., 2022). GIR-Combat therefore does not use MNN alignment as the final correction itself. Instead, it uses MNN-derived cross-batch correspondence as the basis for reference construction, while relying on a ComBat-based model to perform global batch adjustment. In this way, local biological correspondence and model-based global correction are incorporated into a unified framework. Accordingly, the performance of GIR-Combat still depends on the quality of the nearest-neighbor matching, particularly in settings with severe variance heterogeneity, noisy local structure, or weak overlap across batches. However, MNN-derived correspondences are used to support virtual-reference construction rather than directly serving as the final corrected output. This makes the framework less directly dependent on local matching quality than a purely MNN-based correction method.

Using the current batch effect correction vector ${\vec{v}}^{\mathrm{ml}}$ , each sample is corrected for the batch effect. The differences between paired samples are attributed to batch effects, and the differences between nearest neighbor sample pairs provide an estimate of the batch effect. Specifically, ${\vec{v}}^{\mathrm{ml}}$ is calculated based on the element-wise difference in feature profiles between paired samples from the first and second batches. This vector represents the local adjustment required to align biologically comparable samples across batches. This method is based on the MNN framework and pioneers the method of constructing correction vectors by using cross-batch feature profile differences, thereby establishing a batch effect strategy. The value of the sample in the batch to be corrected minus the correction vector is considered to have corrected the batch. Using the current batch effect correction vector ${\vec{v}}^{\mathrm{ml}}$ , perform batch effect correction for each sample to obtain the sample-specific correction vector $\vec{u}(x)$ for samples $\vec{x}$ in batch 2. The calculation formula for $\vec{u}(x)$ is shown in Equations 7, 8, where the parameters in Equation 8 are $\lambda_{1}=0.9,\lambda_{2}=0.1$ . The weights for this linear combination are calculated using the gaussian kernel method W(x,m) where paired samples closer to the target sample have higher weights, while farther paired samples have lower weights. The use of a rank-based distance component in Equation 8 improves robustness to small-scale noise and outlying deviations, thereby stabilizing neighborhood-based weighting in high-variance settings.

To align the dataset with the label subspace of the two batches, the sample vectors ${\vec{E}}^{x}$ are projected onto the batch effect correction vectors ${\vec{v}}^{\mathrm{ml}}$ and shifted to the center of each batch. The calculation method for sample vectors ${\vec{E}}^{x}$ is demonstrated in Equation 9 (Tran et al., 2020).

$$\vec{u}(x)=\frac{\sum_{m}\;{\vec{v}}^{\mathrm{ml}}W(x,m)}{\sum_{m}\;W(x,m)}=\frac{\sum_{m}\;{\vec{v}}^{\mathrm{ml}}e^{-\;\frac{D({\vec{E}}^{x},{\vec{E}}^{m})}{2\sigma^{2}}}}{\sum_{m}\;e^{-\;\frac{D({\vec{E}}^{x},{\vec{E}}^{m})}{2\sigma^{2}}}}$$ (7)

$$D({\vec{E}}^{x},{\vec{E}}^{m})=\lambda_{1}{\left\|{\vec{E}}^{x}-{\vec{E}}^{m}\right\|}^{2}+\lambda_{2}\lim_{p\to \infty }{\left(\sum_{i=1}^{G}{|{{\vec{E}}^{x}}_{i}-{\vec{E}}_{i}^{m}|}^{p}\right)}^{\frac{1}{p}}$$ (8)

$${\vec{E}}^{x}={\vec{E}}^{x}-\vec{u}(x)$$ (9)

Sample l and sample m are considered mutually nearest neighbors only if sample ‘s nearest neighbor list, contains m , and sample m ‘s nearest neighbor list contains l .

As observed from the aforementioned calibration process, the identification of mutually nearest neighbors between the first two batches leads to the generation of sample pair lists, a critical component in the reference batch formation. Given that the computational load of nearest neighbor queries grows exponentially with the size of the dataset, the Annoy approximate nearest neighbor search algorithm (Aumüller et al., 2017) is employed to enhance the efficiency of our approach. Utilizing the Annoy algorithm, the process identifies corresponding mutually nearest neighbor pairs.

The Annoy algorithm recursively divides the sample space and builds multiple binary search trees to establish indexes. Each tree splits the samples in the projected direction through a random hyperplane. Subsequently, the priority queue traversal tree structure is utilized, and the search path is dynamically adjusted based on the distance from the samples to the segmentation hyperplane to efficiently find the mutual nearest neighbor sample pairs. The detailed process can be referred to Supplementary material.

**Figure 1:**
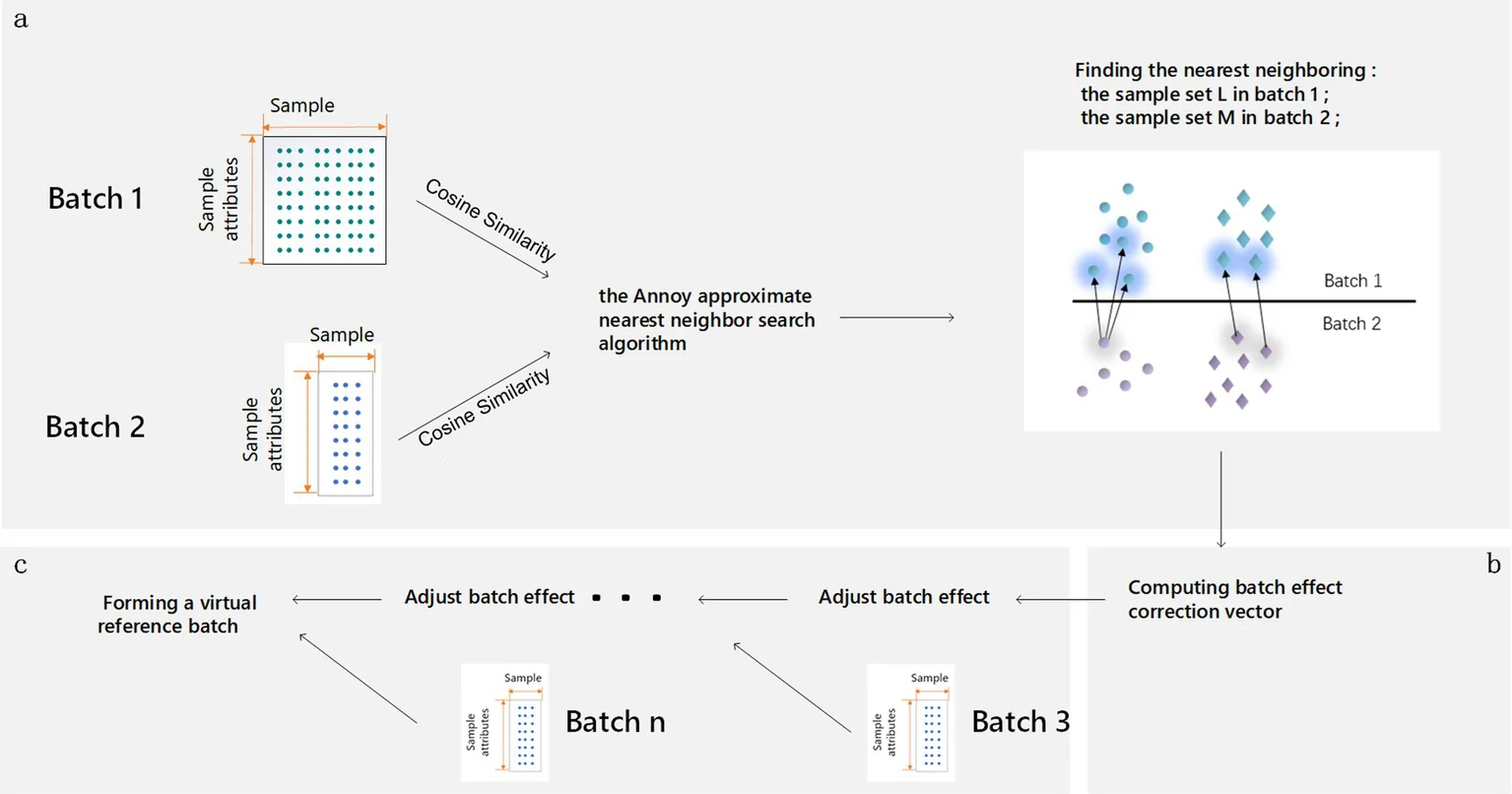
Constructing a virtual reference batch workflow diagram. **(a)** The first two batches identify the mutually nearest neighbors list through the Annoy algorithm, **(b)** the first two batches remove batch effects using batch effect correction vectors. **(c)** Following **(a,b)**, all batches are processed to form a virtual reference batch.

Notes: The challenge arises in the process of forming a virtual reference batch, as previously described, when it is applied directly as the batch effect correction result for data exhibiting large variance. This is because manually specifying a batch as the reference batch may lead to incorrect sample pair matching for samples with different sample labels. If we randomly select a batch as the reference batch for data with large variance, the reference batch is paired with the batch to be calibrated. When samples are incorrectly paired, errors may occur, and the next batch of samples continues to undergo sample pairings with the results of the current batch’s erroneous pairings. The previous error is carried over to the current round, resulting in error accumulation. If not addressed, these accumulated errors can lead to inaccurate calibration results. Assume the following scenario as Figure 2.

For the first two batches of samples in the dataset, the correction vector for each sample is calculated to adjust for batch-specific biases and ensure that they are aligned to a uniform scale. The correction vectors calculated in the first two batches are applied to the third batch of samples to correct and adjust them. Repeat the process for each remaining batch. The correction vector for the current batch is calculated each time and applied to the correction for subsequent batches. All the sample data after batch correction are summarized to form a matrix $S_{\mathrm{ijg}}$ . The complete process for constructing a virtual reference batch is illustrated in Figure 1.

**Figure 2:**
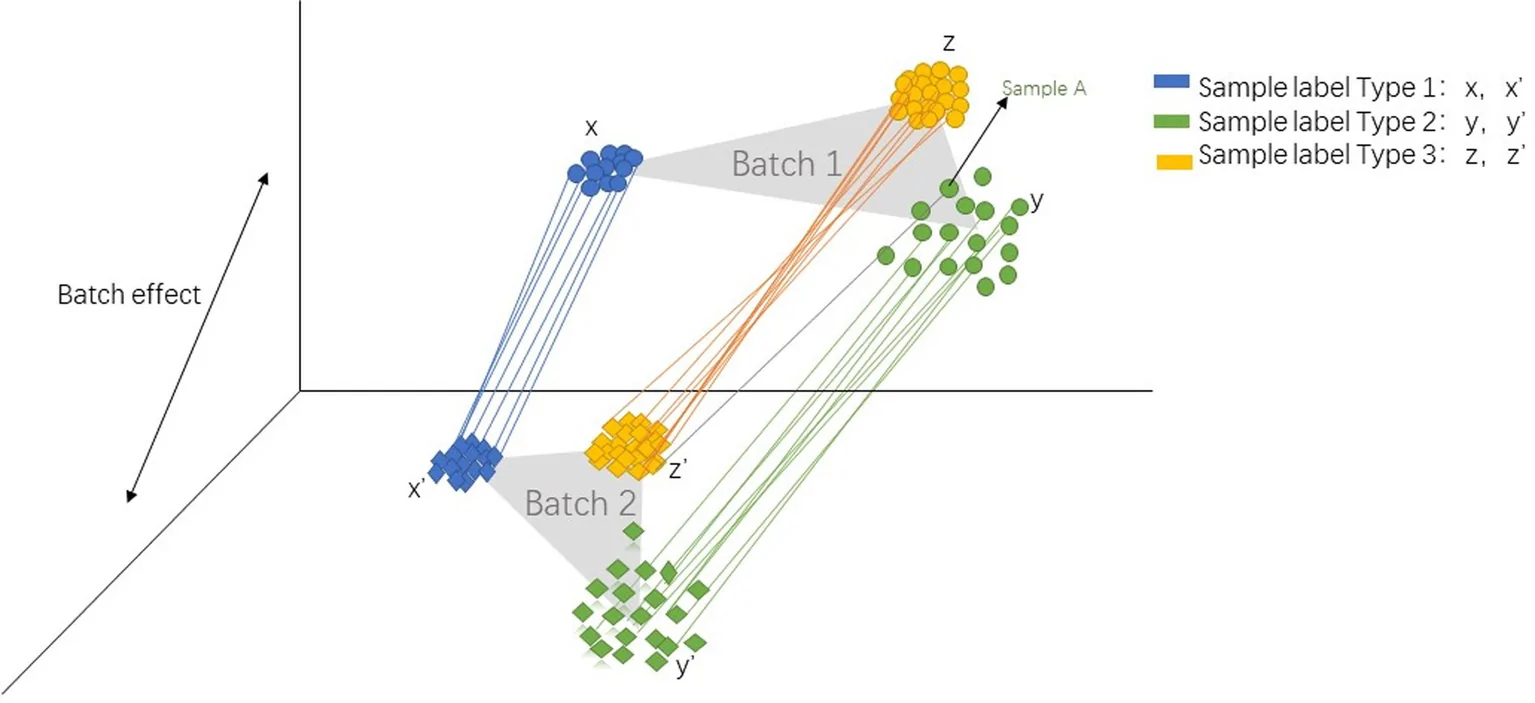
Schematic illustration of the erroneous scenario involving the formation of Mutual Nearest Neighbors sample pairs list across two batches.

The dataset was comprised of two batches, each containing three label types. Significant differences between the label types led to notable variance in expression values, particularly for label type 2, relative to the other label types. Assuming that label type 2 in batch 1 had a sample A. If in batch 2, sample A can be paired. During the search for mutual nearest neighbor pairs, the large variance of label type 2 increases the likelihood of incorrect pair matching for sample A. This may result in the mismatch of k nearest neighbors with another label type in batch 2, leading to errors in the calculation of the correction vector for sample A in batch 1. If in batch 2, there are no paired samples of sample A. This performs a local approximation, i.e., a linear combination of the correction vectors of samples with successful pairings around sample A to calculate the correction vector of sample A. The closer the samples are to sample A the greater the weights assigned in the process of linear combination. However, the large variance property of the characteristics of label type 2 corresponding to sample A is surrounded by other label types with a high probability in the process of our approach to constructing reference batches performing the local approximation. In such cases, using a linear combination of correction vectors obtained from samples of other sample types to correct the distance between batch 1 and batch 2 would inevitably be incorrect.

It should be noted that this method requires building a reference batch for each batch, which must contain at least one same label. If this condition is not met, the method cannot be applied directly. In this case, we suggest clustering other variables to generate pseudo-tags that achieve a similar effect and allow reference batches to be built using our method. In addition, future work could explore adapting algorithms to handle single-label batches by leveraging global information from other batches to compensate for the lack of label diversity.

#### 2.2.2 Add the reference batch to GIR-combat model

The matrix $S_{\mathrm{ijg}}$ formed by the reference batch in the previous step are merged into the original matrix $Y_{\mathrm{ijg}}$ to form the new matrix $H_{\mathrm{ijg}}$ to be next corrected. In the subsequent step, the reference batch matrix $S_{\mathrm{ijg}}$ obtained from the previous step and the original matrix $Y_{\mathrm{ijg}}$ are merged column-wise, based on the shared feature of having the same sample attribute dimensions. This results in a new matrix $H_{\mathrm{ijg}}$ for further batch effect correction, where the sample attribute dimensions remain unchanged, and the sample dimensions are doubled. The specific formula can be expressed as $H_{\mathrm{ijg}}=[Y_{\mathrm{ijg}}|S_{\mathrm{ijg}}]$ , add setting all the columns corresponding to the newly formed reference virtual batch b in the batch design matrix $G_{\mathrm{ij}}$ to 1. (As in the 2.1 Ref-Combat part, ‘m’ has the same meaning, representing the total number of batches. For convenience, it is denoted here as the $b^{\mathrm{th}}$ batch. Here, define b=m+1 .) Based on the reformulated ComBat expression from presented in the article introducing the reComBat model by Adamer et al. (2022), $H_{\mathrm{ijg}}$ , which means the value of the $g^{\mathrm{th}}$ attribute in the $j^{\mathrm{th}}$ sample of the $i^{\mathrm{th}}$ batch, is defined as this equivalent Equation 10:

$$H_{\mathrm{ijg}}=\alpha_{\mathrm{bg}}+D_{\mathrm{ij}}F_{\mathrm{bg}}+G_{\mathrm{ij}}U_{\mathrm{big}}+\varepsilon_{\mathrm{ijg}}\;\;\;\text{where}\;\varepsilon_{\mathrm{ijg}}∼N(0,\sigma_{\mathrm{bg}}^{2})$$ (10)

Where $\alpha_{\mathrm{bg}}$ represents the average value of the $g^{\mathrm{th}}$ attribute in the virtual reference batch ( b ), $D_{\mathrm{ij}}F_{\mathrm{bg}}$ is the mainly informative part of the profile matrix, $D_{\mathrm{ij}}$ is the bio design matrix, which contains information on the type of biology corresponding to each sample, $F_{\mathrm{bg}}$ is the coefficient matrix of information on the biological types corresponding to each sample. $G_{\mathrm{ij}}U_{\mathrm{big}}$ is the part of the batch information in the matrix, $G_{\mathrm{ij}}$ is the information on the corresponding batch for each sample, $U_{\mathrm{big}}$ is the coefficient matrix of the batch information corresponding to each sample, $\varepsilon_{\mathrm{ijg}}$ is the random errors caused by measurements when obtaining data.

Based on Equation 10, the process of eliminating batch effects for $H_{\mathrm{ijg}}$ is as follows: Set the corresponding column of the newly formed virtual batch of sample in the batch design matrix $G_{\mathrm{ij}}$ to all 1 s.

Following the Ref-Combat process as depicted in Equation 2, the standardized $H_{\mathrm{ijg}}$ with the estimated batch effects removed is calculated as $Z_{\mathrm{ijg}}$ . Set the initial parameters of the prior distribution, where the initial parameter $\;\gamma_{\mathrm{big}}$ is calculated according to Equation 11.

$${Z_{\mathrm{ijg}}}^{T}Z_{\mathrm{ijg}}\hat{\gamma_{\mathrm{big}}}={Z_{\mathrm{ijg}}}^{T}Z_{\mathrm{ijg}}$$ (11)

The initial parameter $\delta_{\mathrm{big}}^{2}$ can be calculated from the row variance of each batch of $Z_{\mathrm{ijg}}$ . Subsequently, the mean parameter $\;\gamma_{\mathrm{big}}$ and the variance parameter $\delta_{\mathrm{big}}$ of the normal distribution to be fitted to $Z_{\mathrm{ijg}}$ are calculated iteratively, employing the same EM algorithm as in the original Ref-Combat.

Row values of 0 and 1 were assigned to the virtual reference batches of $\;\gamma_{\mathrm{big}}$ and $\delta_{\mathrm{big}}$ . This indicates that the reference batches were considered idealized data without batch effects and that all matrix expression values were adjusted towards the reference batches, using them as a benchmark. This confirms the theory’s validity that the corrected batch is used as the reference batch for secondary correction. In accordance with the Ref-Combat process, the results obtained after removing the batch effects are denoted as $H_{\text{correct}}$ , which is calculated according to Equation 12.

$$H_{\text{correct}}=Z_{\mathrm{ijg}}-N(\;\gamma_{\mathrm{big}},\delta_{\mathrm{big}}^{2})+({\hat{\alpha }}_{\mathrm{bg}}+D_{\mathrm{ij}}{\hat{F}}_{\mathrm{bg}})$$ (12)

Finally, the columns corresponding to the virtual reference batch are removed from the corrected expression matrix $H_{\text{correct}}$ , resulting in the formation of $Y_{\text{correct}}$ . Through Spherical Kmeans clustering (SPKM), our model can handle datasets without explicitly labeled sample types. The aim of this step is to restore as much of the original sample label type information as possible. Based on the theoretical basis that the cosine relative distances of different label types are preserved across batches (Kim et al., 2019), We make SPKM clustering based on the cosine correlation for the entire dataset across batches (Hornik et al., 2012), which allows us to obtain the labels of the different label types to which the samples in this matrix to be corrected belong.

Algorithm 1 is the overall algorithm flow of GIR-Combat. The input of the algorithm is the matrix $Y_{\mathrm{ijg}}$ , the sample batch indicator vector C, and the output is the corrected matrix $Y_{\text{correct}}$ . The SPKM function in Step1 performs SPKM clustering on the matrix to be corrected to obtain the label type vector E of the samples. If the dataset has already provided a vector of sample label types, this step can be skipped. The Form_Refbatch function in Step2 means the process of forming a reference batch. Form_Refbatch groups samples in $Y_{\mathrm{ijg}}$ according to their labels in E. It creates a reference subset by combining samples that represent the central tendency of each cluster in E. Step3 means the reference batch combines the columns of the correction matrix to construct a new correction batch indicator vector $D_{\mathrm{ij}}$ and obtain the target matrix for linear modeling. The Empirical_Bayes_Estimates function in the fourth line means $H_{\text{correct}}$ is obtained through empirical Bayesian estimation, following the same procedure as in Ref-Combat. The Remove_Refbatch function in the fifth line means the reference batch is removed from $H_{\text{correct}}$ to obtain the final corrected result $Y_{\text{correct}}$ .

Algorithm 1: GIR-Combat Algorithm.
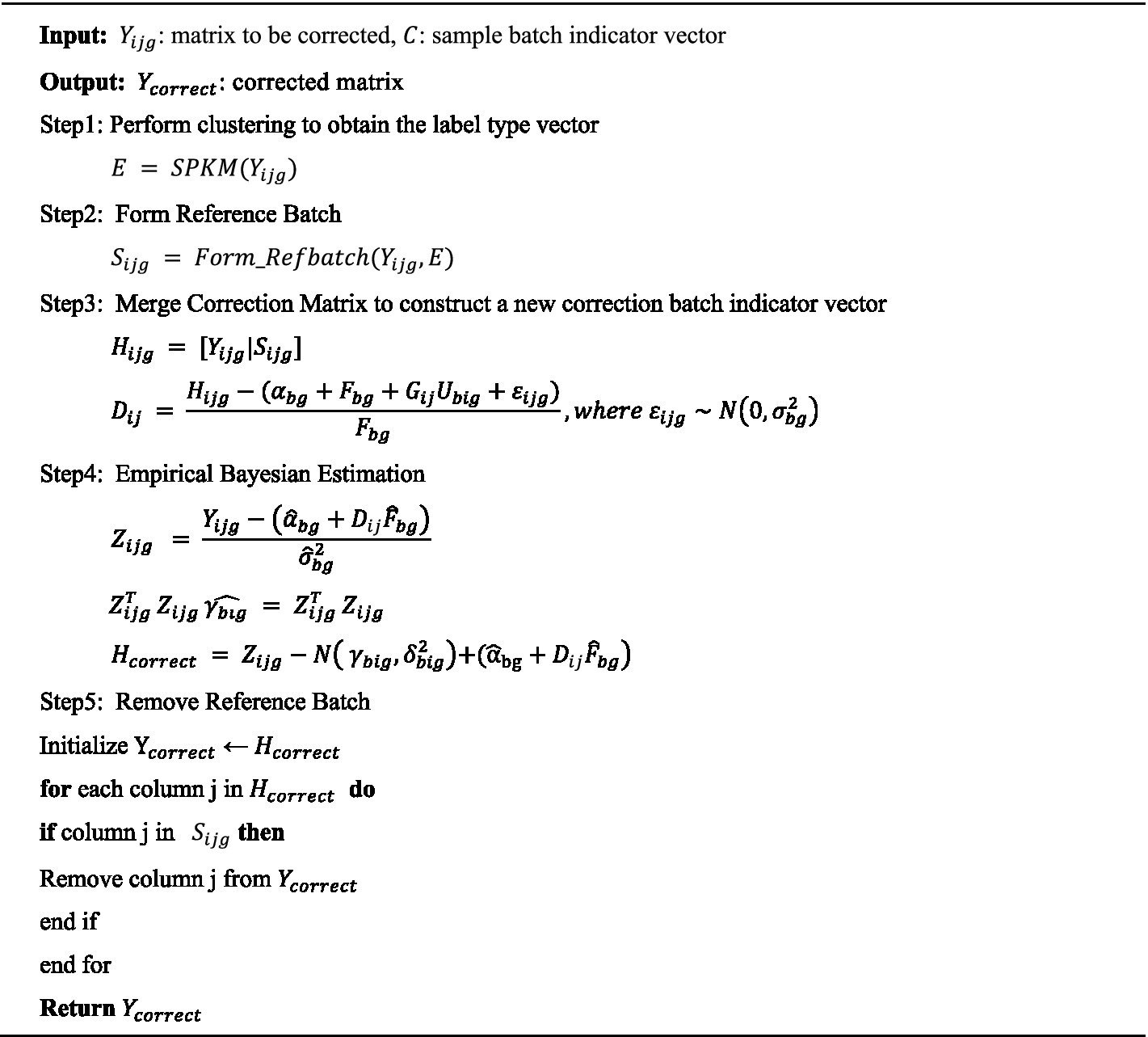

## 3 Results

We evaluated GIR-Combat on both the simulation dataset and multiple real datasets. Representative examples include the colorectal cancer gene expression dataset, which provides a high-variance bulk expression setting; the human dendritic cell dataset, which captures compositional imbalance and high variance; the mouse HSPC dataset, which reflects partial lineage overlap; the Murine Cell Atlas, which represents a cross-platform integration setting; and the human PBMC dataset, which reflects protocol-driven batch effects. Together, these datasets span a range of integration scenarios relevant to reference-based batch correction, including high variance, compositional imbalance, incomplete overlap across batches, cross-platform variation, and heterogeneous technical structure.

To systematically assess the performance of GIR-Combat, we first conducted three simulation experiments designed to isolate the effects of variance structure and batch composition. We then validated the framework on multiple public datasets exhibiting distinct integration challenges. Batch correction performance was evaluated using dimensionality reduction visualizations and relevant evaluation metrics. GIR-Combat was compared with Combat, MNN, Harmony, Seurat (Satija et al., 2015), and LIGER (Liu et al., 2020) across both simulated and the real datasets. Results demonstrate that GIR-Combat provides robust batch correction in high-variance settings while preserving biologically meaningful structure.

### 3.1 Simulation studies

The simulation experiments were designed to evaluate GIR-Combat under progressively more challenging correction scenarios. Each simulated dataset contained three batches and three biological groups, with scenario-specific differences in variance structure and batch composition. A balanced dataset contains all biological groups in every batch. On the other hand, a compositionally imbalanced dataset is one where at least one of the batches lacked at least one biological group.

**Figure 3:**
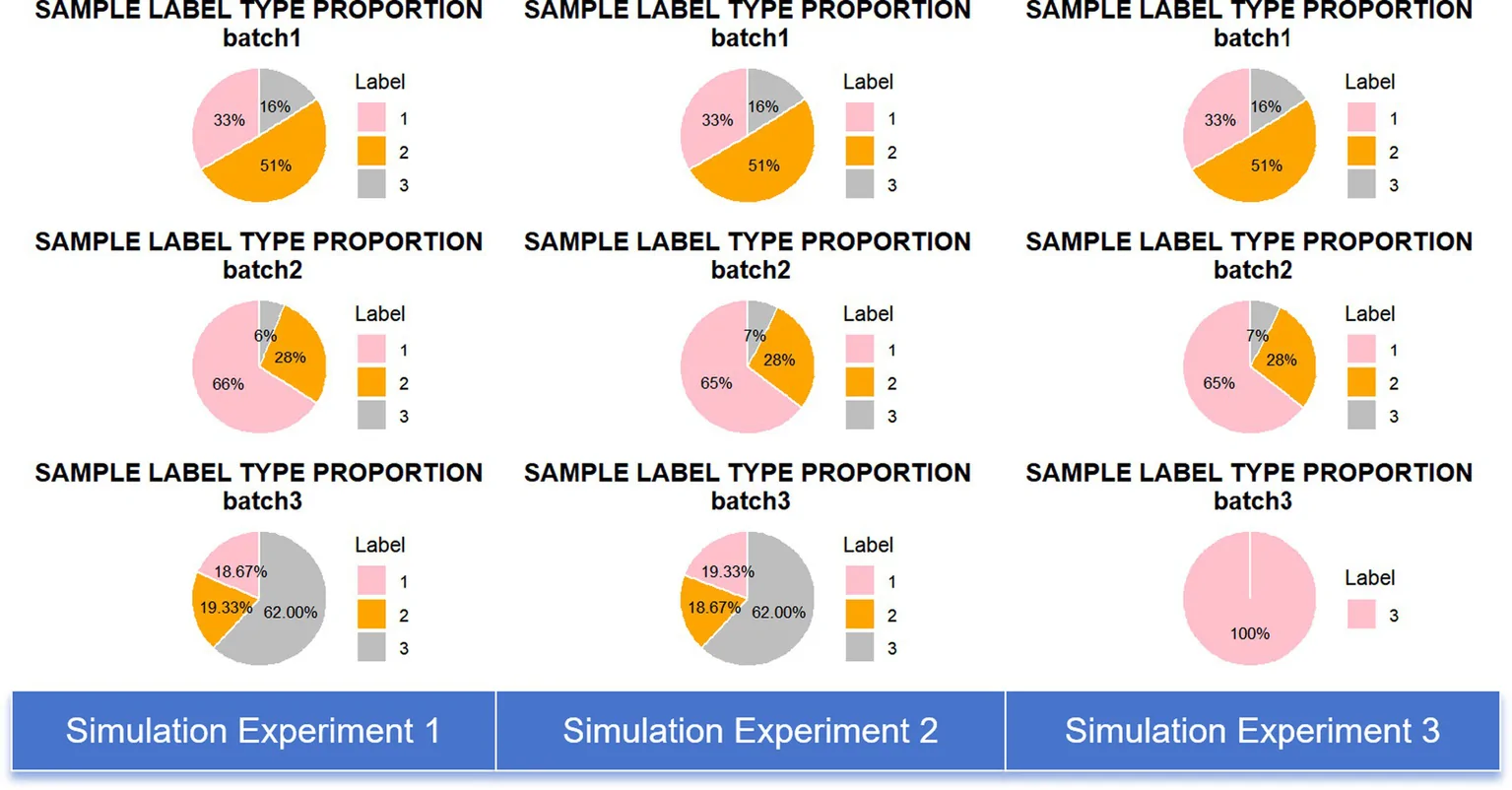
Proportion of sample label composition for each batch in the simulated dataset.

Three simulation settings were considered. Experiment 1 represented a balanced scenario with comparable variance across biological groups. Experiment 2 introduced elevated variance in one biological group while maintaining balanced batch composition. Experiment 3 combined elevated variance with compositional imbalance, thereby representing the most challenging condition for reference-based correction. Figure 3 illustrates the composition of sample labels encompassed within each simulated dataset. Table 1 presents the mean and variance for the three sets of simulation experiment configurations.

**Table 1:** The mean and variance for the given batch on the simulated datasets.

Dataset	Characteristic	Batch 1	Batch 2	Batch 3
Sim 1	Mean of the x-coordinate in the low-dimensional space	0	5	5
	Mean of the y-coordinate in the low-dimensional space	5	5	0
	Variance of the x-coordinate in the low-dimensional space	1	1	1
	Variance of the y-coordinate in the low-dimensional space	1	1	1
Sim 2	Mean of the x-coordinate in the low-dimensional space	0	5	5
	Mean of the y-coordinate in the low-dimensional space	5	5	0
	Variance of the x-coordinate in the low-dimensional space	1	3	1
	Variance of the y-coordinate in the low-dimensional space	1	3	1
Sim 3	Mean of the x-coordinate in the low-dimensional space	0	5	5
	Mean of the y-coordinate in the low-dimensional space	5	5	0
	Variance of the x-coordinate in the low-dimensional space	1	3	1
	Variance of the y-coordinate in the low-dimensional space	1	3	1

#### 3.1.1 The UMAP visualization method by density map

**Figure 4:**
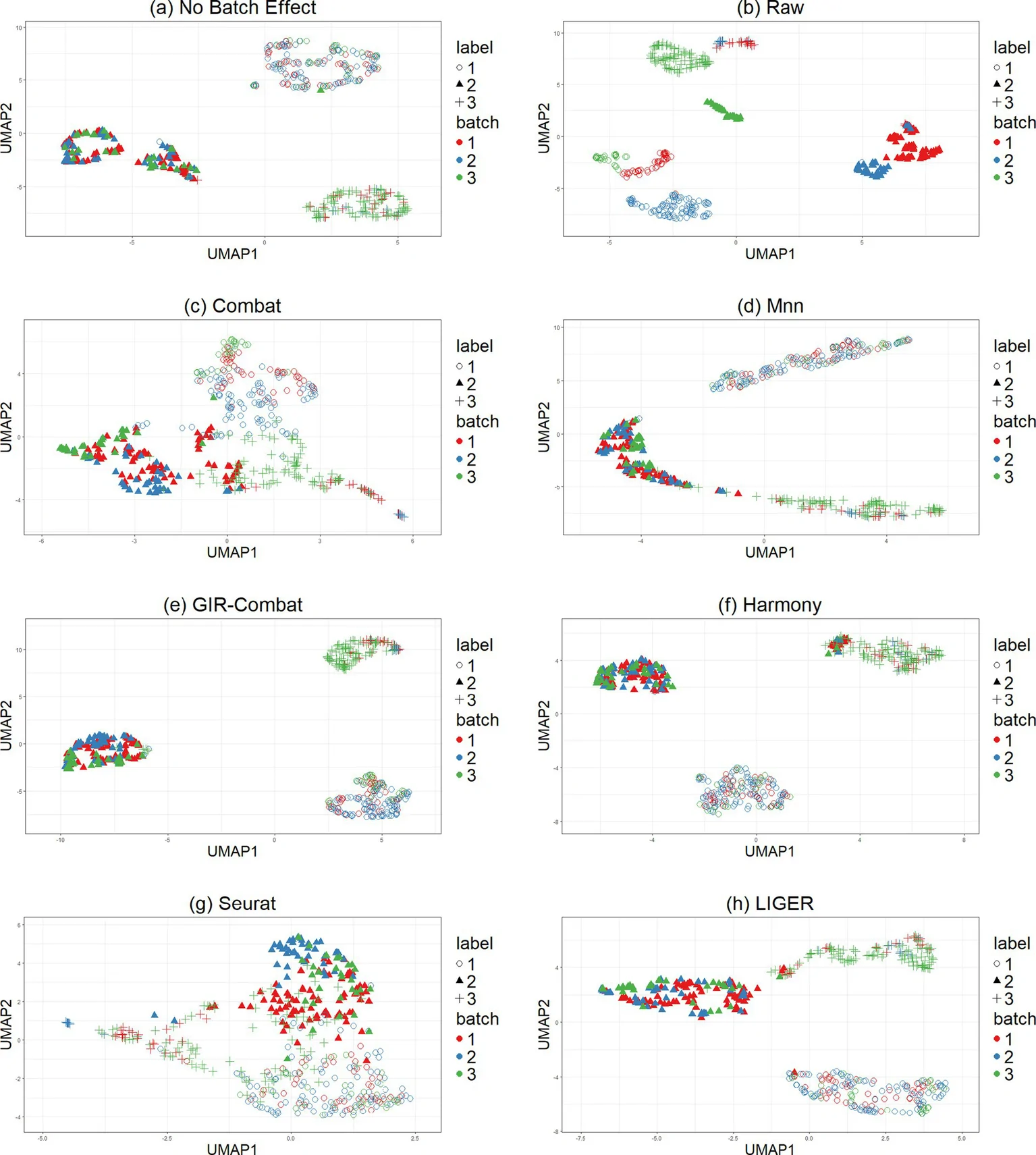
UMAP plots of density visualization of simulated data before and after batch effect correction of the simulation experiment 1. **(a)** No batch data; **(b)** Batch data; **(c)** Batch data corrected by the Combat model; **(d)** Batch data corrected by the MNN; **(e)** Batch data corrected by the GIR-Combat model; **(f)** Batch data corrected by the Harmony; **(g)** Batch data corrected by the Seurat; and **(h)** Batch data corrected by the LIGER. Within the graphical representations, the distinct colors and shapes of the sampling points correspond to their respective batch and sample label classification.

**Figure 5:**
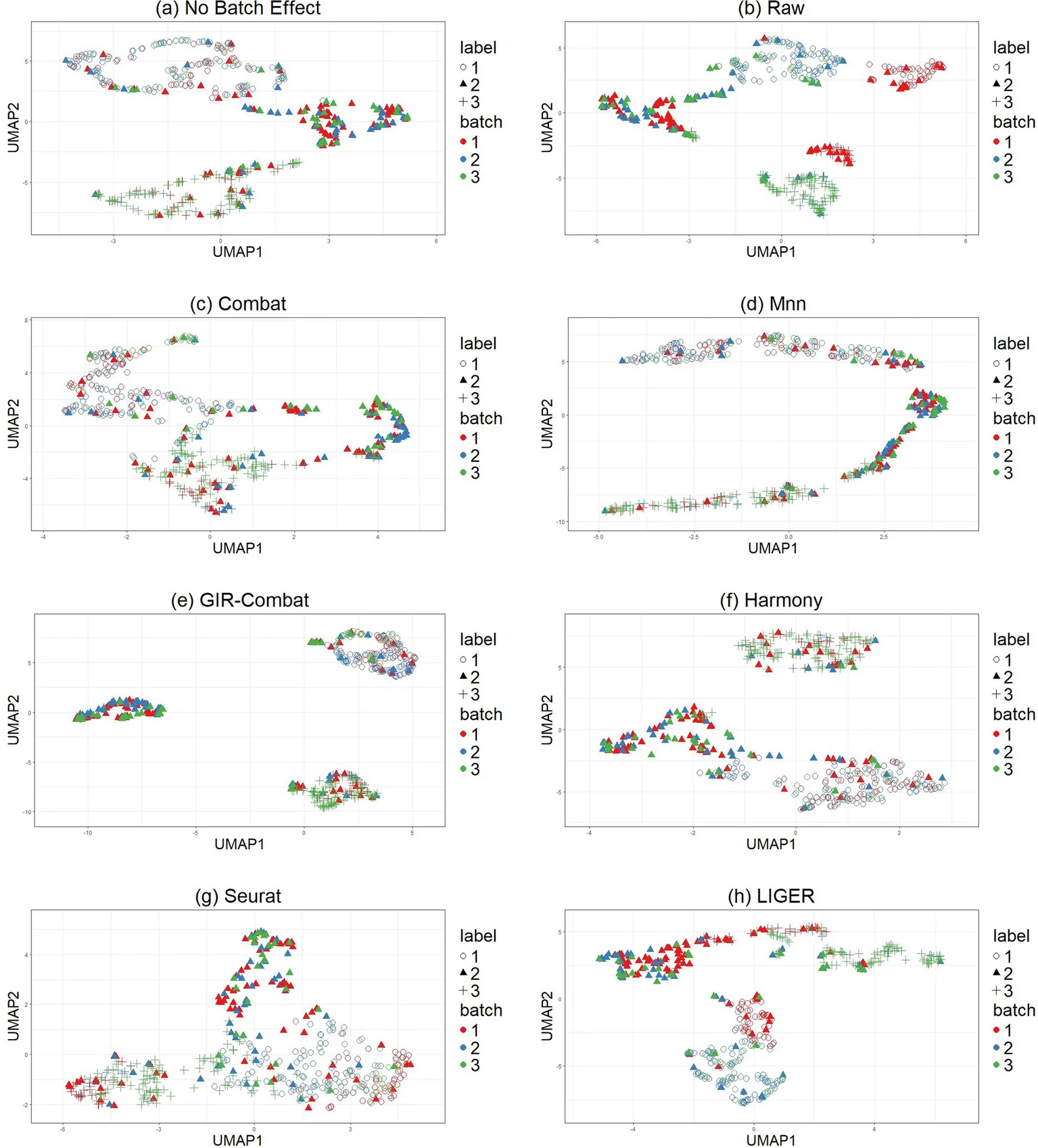
UMAP plots of density visualization of simulated data before and after batch effect correction of the simulation experiment 2. **(a)** No batch data, **(b)** Batch data, **(c)** Batch data corrected by the Combat model, **(d)** Batch data corrected by the MNN, **(e)** Batch data corrected by the GIR-Combat model, **(f)** Batch data corrected by the Harmony, **(g)** Batch data corrected by the Seurat and **(h)** Batch data corrected by the LIGER.

**Figure 6:**
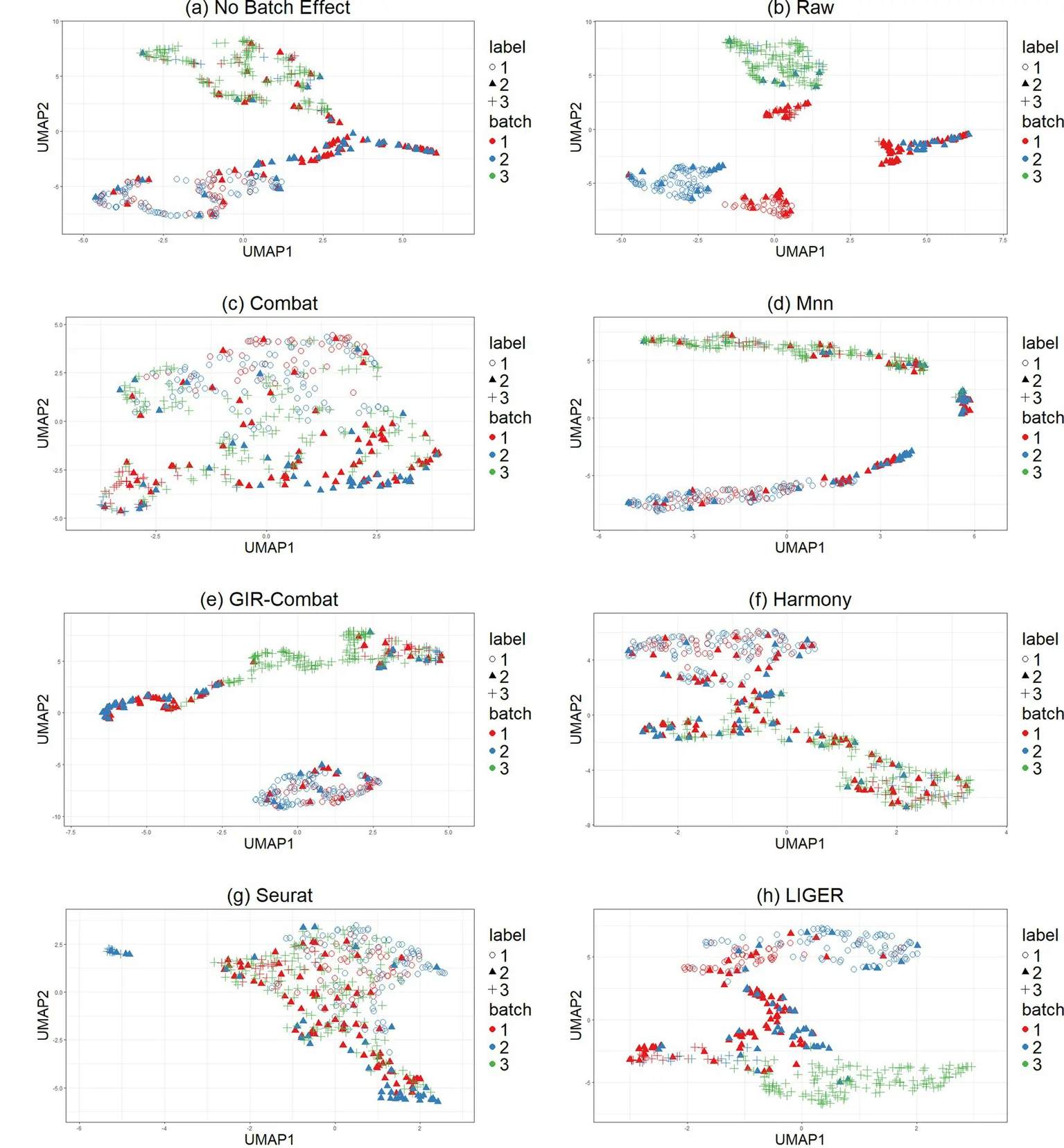
UMAP plots of density visualization of simulated data before and after batch effect correction of the simulation experiment 3. **(a)** No batch data, **(b)** batch data, **(c)** batch data corrected by the Combat model, **(d)** batch data corrected by the MNN, **(e)** batch data corrected by the GIR-Combat model, **(f)** batch data corrected by the Harmony, **(g)** batch data corrected by the Seurat and **(h)** batch data corrected by the LIGER.

Batch integration performance was first assessed visually using UMAP. Data without batch effects, data with batch effects, data corrected for Combat, MNN, GIR-Combat, Harmony, Seurat and LIGER were visualized using the UMAP (McInnes et al., 2018) visualization method. In the absence of batch effects, samples belonging to the same sample label exhibited a consistent distribution pattern, indicating well-preserved biological structure without technical variation (Figures 4a, 5a, 6a). Before batch effect correction, samples within the same batch clustered together due to batch effects, leading to an artificial grouping dominated by batch assignment rather than biological similarity (Figures 4b, 5b, 6b). After applying batch correction, differences in performance among the methods became evident. Combat mitigated some batch effects but failed to completely separate different sample labels, indicating an incomplete removal of technical variation (Figures 4c, 5c, 6c). Similarly, MNN correction further reduced batch-induced shifts but did not fully resolve inter-sample label distinctions, leaving residual batch-related clustering (Figures 4d, 5d, 6d). While Harmony performed reasonably well in the first experiment, its effectiveness declined in the other two scenarios, suggesting that its performance is sensitive to dataset characteristics (Figures 4f, 5f, 6f). The different sample label cannot be completely separated after Seurat and LIGER correction (Figures 4g,h, 5g,h, 6g,h). GIR-ComBat aligns samples across batches based on their biological similarity, minimizing the influence of technical artifacts. As a result, after correction, samples belonging to the same biological group should cluster together regardless of their original batch, while distinct groups should remain separated. GIR-Combat not only successfully corrected batch effects but also maintained clear separation among different sample labels, ensuring that biological structure was preserved while batch effects were eliminated (Figures 4e, 5e, 6e).

#### 3.1.2 Variance calculation: linear model per variable

**Figure 7:**
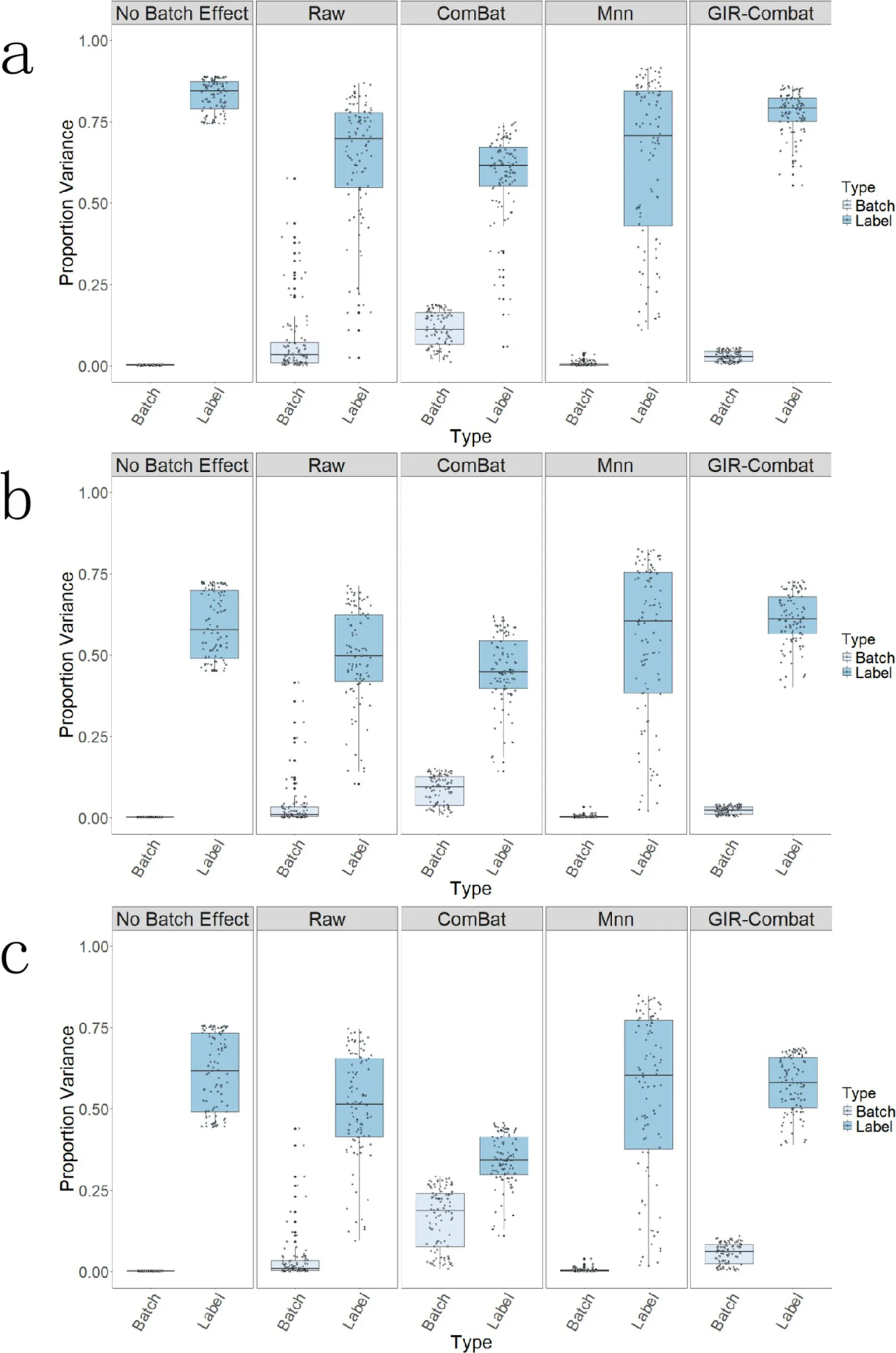
The variance explained plot for the fitted linear mixed model before and after batch effect correction of the simulation experiment: **(a)** Simulation experiment 1, **(b)** Simulation experiment 2, and **(c)** Simulation experiment 3.

To quantify the attribution of batch effects and biological labels on variation, we performed variance decomposition using a linear mixed model. The percentage of variance explained by fitting the linear mixed model is calculated and extracted from the variance due to batch and sample label by using the fitExtractVarPartModel function in the variancePartition package in R (Hoffman and Schadt, 2016). Figure 7 depicts the composition of variance explained for each simulated dataset. Ideally, a successful batch correction method should reduce the variance attributed to batch effects while maintaining the diversity associated with biological labels at a level comparable to the original data. Results demonstrate that after applying GIR-Combat, the variance contribution from batch is significantly reduced, indicating effective removal of batch effects, while the variance associated with labels remains stable, thereby preserving meaningful biological differences without distortion. This supports the effectiveness of GIR-Combat in removing technical artifacts while preserving critical biological information in a biologically meaningful manner.

#### 3.1.3 Average silhouette width (ASW)

The performance of the batch effect correction method is evaluated using cluster analysis in two main ways: the degree of batch mixing of samples and the purity of sample label types. To apply ASW on batch quantification, it is used to assess the stability of batch clusters (Büttner et al., 2019; Luecken et al., 2022). Our model outperforms other methods in terms of the internal clustering evaluation index, $F1_{\mathrm{ASW}}$ (Rousseeuw, 1987). $F1_{\mathrm{ASW}}$ represents a measure where lower $\mathrm{AS}W_{\text{batc}h}$ scores indicate better mixing of different batch of samples, while higher $\mathrm{AS}W_{\text{label}}\;$ scores signify enhanced higher purity of sample labels. For example, in simulation experiment 3, the $F1_{\mathrm{ASW}}$ scores for GIR-Combat at 0.5558891 and Harmony at 0.556765, while ComBat at 0.512901, MNN at 0.5433263, Uncorrected data at 0.5253156, Seurat at 0.52288, and LIGER at 0.5078176, indicating that GIR-Combat and Harmony maintained the highest clustering metrics after batch effect correction and the best batch effect correction. At the same time, its performance is closest to the data with No Batch Effect. Table 2 displays the $F1_{\mathrm{ASW}}$ values before and after batch effect correction for the three sets of simulation experiments, utilizing various correction methods. In this table, Uncorrected indicates that there is a batch effect but no correction, and No Batch Effect indicates that there is no batch effect.

**Table 2:** The ASW score of the batch effects correction methods of the simulation experiments.

Methods	Dataset	GIR-Combat	MNN	Combat	Uncorrected	No Batch Effect	Harmony	Seurat	LIGER
$F1_{\mathrm{ASW}}$ score	Sim 1	0.6058477	0.5858406	0.5693649	0.5689675	0.6075813	0.63340	0.57883	0.61151
	Sim 2	0.5785856	0.5671041	0.5398617	0.5446897	0.5914878	0.5895026	0.5737433	0.5517282
	Sim 3	0.5558891	0.5433263	0.512901	0.5253156	0.5780207	0.556765	0.52288	0.5078176

The silhouette width of sample is defined in Equation 13:

$$F1_{\mathrm{ASW}}=\frac{2*(1-\mathrm{AS}W_{\text{batch}})*(\mathrm{AS}W_{\text{label}})}{1-\mathrm{AS}W_{\text{batch}}+\mathrm{AS}W_{\text{label}}}$$ (13)

### 3.2 Real dataset studies

We next evaluated GIR-Combat on multiple public datasets representing distinct real-world integration challenges. These datasets were selected to complement the simulation experiments by assessing performance in settings characterized by high variance, compositional imbalance, partial cross-batch overlap, cross-platform heterogeneity, and protocol-driven batch effects.

#### 3.2.1 Colorectal cancer gene expression datasets

The colorectal cancer gene expression datasets were included as a representative high-variance bulk expression benchmark, which is presented in the article by Nyamundanda, Gift, et al. in 2017 (Nyamundanda et al., 2017). The dataset contains two types of samples, normal and cancer. Two gene expression datasets (GSE18088 (Gröne et al., 2011) and GSE23878 (Uddin et al., 2011); Affymetrix GeneChip® Human Genome U133 Plus 2.0 Array) comprise 52 and 58 samples, respectively. The cancer samples were trimmed to align with the desired characteristics of the datasets. Among the 52 samples derived from GSE18088, all were classified as CRCs (colorectal cancer). In contrast, within the 58 samples obtained from GSE23878, 24 samples were derived from normal tissues, while the remaining 34 samples were categorized as tumor samples, of which 24 were used for the experiment. The dataset description is shown in Table 3. Furthermore, since the dataset corresponds to samples originating from either normal tissue or tumor samples, data preprocessing through clustering is not required.

**Table 3:** The sample contained in each batch.

	Normal	Cancer
Batch 1	0	52
Batch 2	24	24

**Figure 8:**
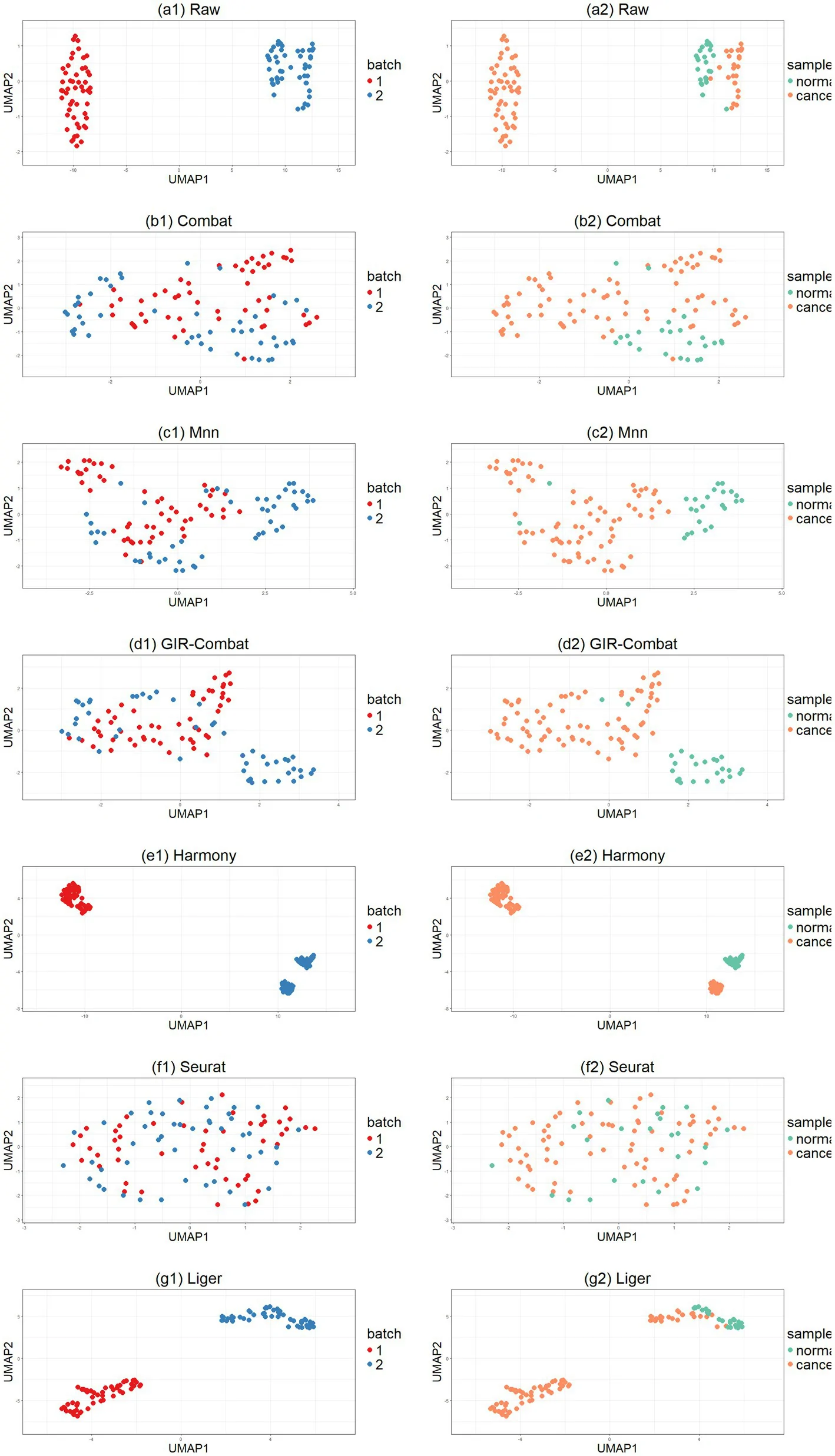
UMAP plots of density visualization of data before and after batch effect correction on colorectal cancer gene expression datasets. **(a)** Batch data, **(b)** batch data corrected by the Combat model, **(c)** batch data corrected by the MNN, **(d)** batch data corrected by the GIR-Combat model, **(e)** batch data corrected by the Harmony, **(f)** batch data corrected by the Seurat, and **(g)** batch data corrected by the LIGER. In each sub-graph, for example, a1 represents the UMAP plots of the distribution of batch data by batch, and a2 represents the UMAP plots of the distribution of batch data by traits.

**Figure 9:**
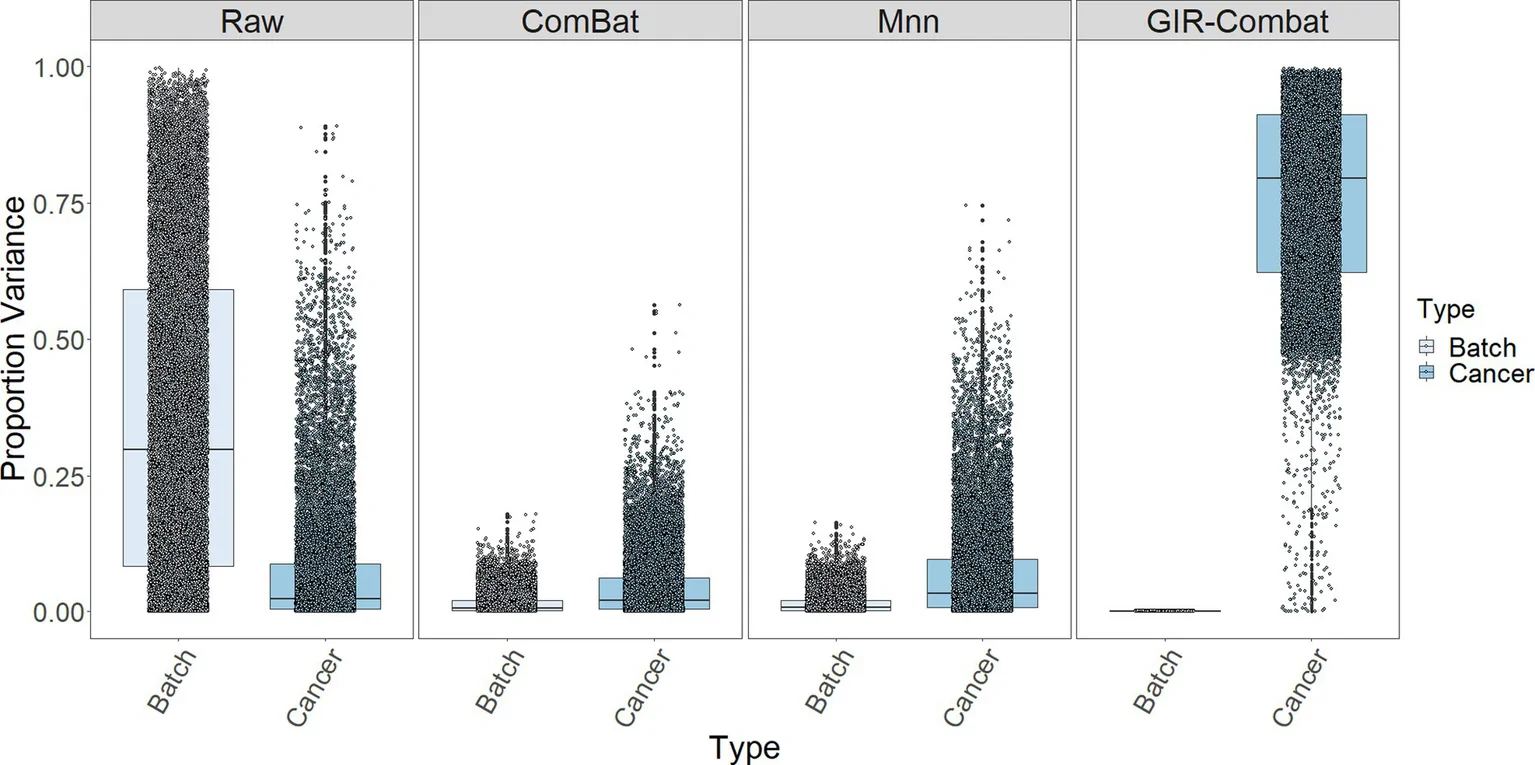
The variance explained plot for the fitted linear mixed model before and after batch effect correction on colorectal cancer gene expression datasets.

Table 3 provides a general overview of this dataset, supporting the characterization that GIR-Combat performs well for large variances. As shown in Table 4, across all batches, the size of the variance of the normal and cancer sample can be ordered as follows: The variance of cancer sample > The variance of normal sample. Figure 8 illustrates the UMAP plots of the colorectal cancer gene expression datasets before and after batch effect correction. Figure 9 depicts the composition of variance explained for this real dataset. Table 5 displays the $F1_{\mathrm{ASW}}$ values before and after batch effect correction for the colorectal cancer gene expression datasets.

**Table 4:** The variance of each batch in the dataset.

	The variance of normal sample	The variance of cancer sample
Batch 1	0	2.01722
Batch 2	2.0*28*266	2.040668

**Table 5:** The ASW score of the correction methods.

Methods	GIR-Combat	MNN	Combat	Uncorrected	Harmony	Seurat	LIGER
$F1_{\mathrm{ASW}}$ score	0.574921	0.530964	0.5222124	0.3968255	0.505118	0.500857	0.449184

#### 3.2.2 Human dendritic cells datasets

The human dendritic cell (DC) dataset was selected as a representative single-cell benchmark characterized by both compositional imbalance and substantial variance across cell populations. Villani et al. (2017) generated this dataset from four cell populations (CD1C dendritic cells, CD141 dendritic cells, plasmacytoid dendritic cells (pDC), and double-negative cells). Each population was sorted and sequenced across two experimental batches using Smart-Seq2. This dataset captures biological variation between distinct immune cell types along with batch-induced technical effects. The data were preprocessed by normalizing each library to the median transcript count of all cells and then log-transformed for expression values. Both batches included pDCs and double-negative cells, each with a biologically similar cell type (CD141 and CD1C cells, respectively). The experimental results show that our model effectively corrects batch effects while preserving the separation between cell types.

**Figure 10:**
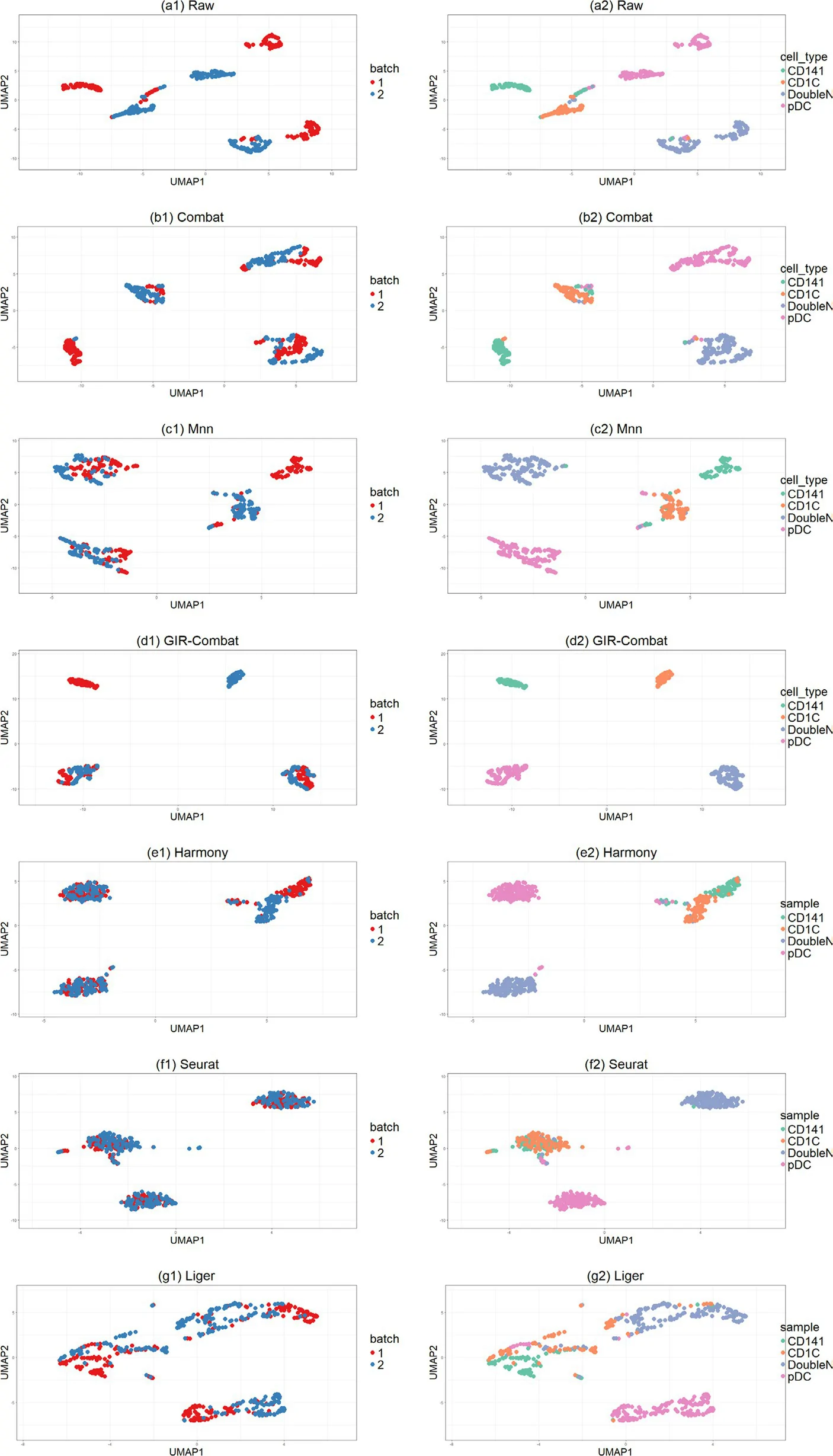
UMAP plots of density visualization of data before and after batch effect correction on Human dendritic cells datasets. **(a)** Batch data, **(b)** Batch data corrected by the Combat model, **(c)** Batch data corrected by the MNN, **(d)** Batch data corrected by the GIR-Combat model, **(e)** Batch data corrected by the Harmony, **(f)** Batch data corrected by the Seurat and **(g)** Batch data corrected by the LIGER. In each subgraph, for example, a1 represents the UMAP plots of the distribution of batch data by batch, and a2 represents the UMAP plots of the distribution of batch data by traits.

**Figure 11:**
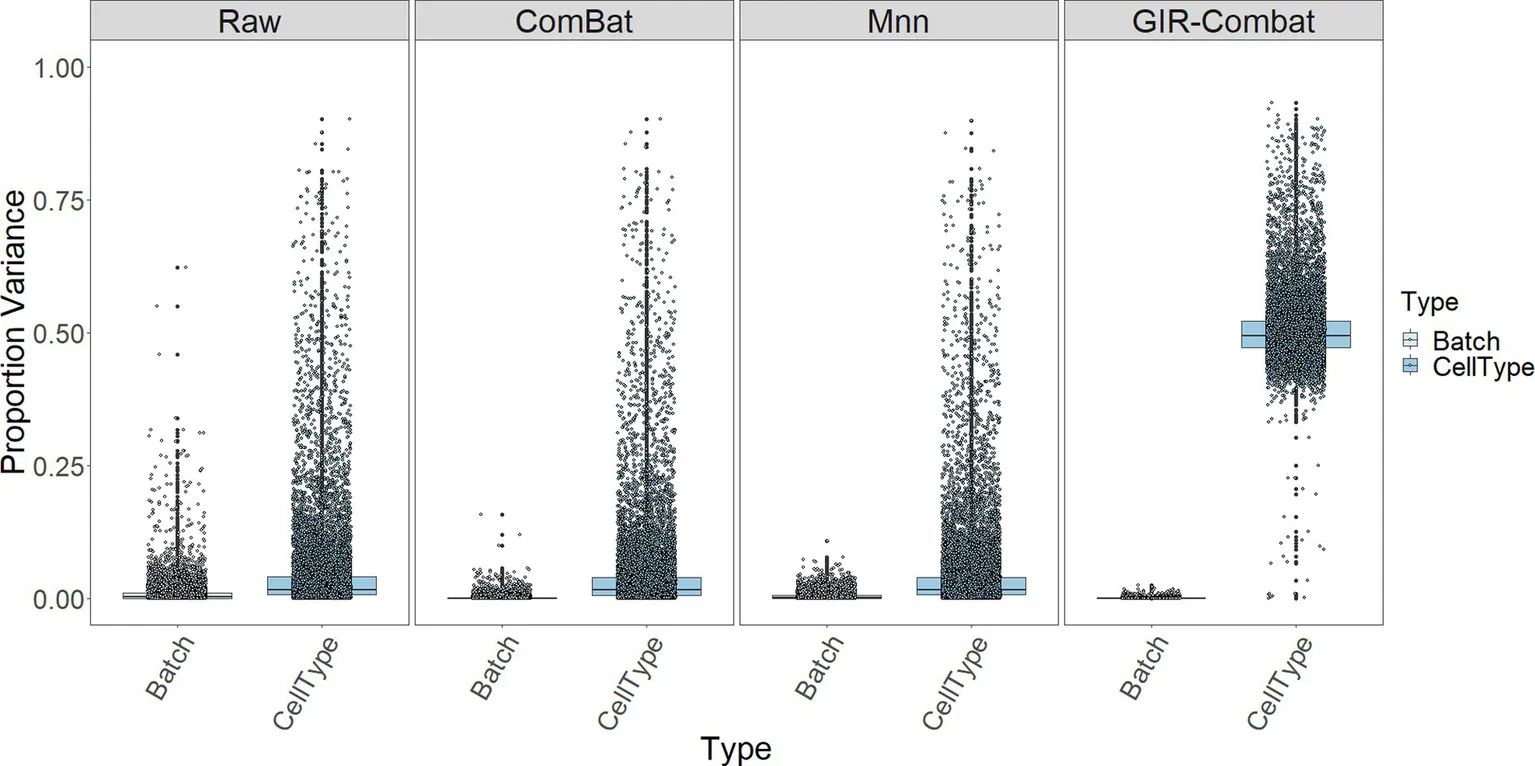
The variance explained plot for the fitted linear mixed model before and after batch effect correction on human dendritic cells datasets.

Table 6 provides a comprehensive summary of the dataset, supporting the characterization that our correction method effectively rectifies the large variances and imbalances specifically addressed by the approach. As demonstrated in Table 7, the magnitude for distinct cell types, as observed across all batches, can be sequentially arranged in the following inequality: the variance of pDC > the variance of DoubleNeg > the variance of CD141 or the variance of CD1C. Figure 10 presents the UMAP visualizations of the human dendritic cells datasets, both prior to and subsequent to the application of batch effect correction. Figure 11 portrays the composition of variance elucidated for this dataset. Meanwhile, Table 8 enumerates the $F1_{\mathrm{ASW}}$ values for the datasets before and after the implementation of batch effect correction.

**Table 6:** The cell numbers contained in each batch.

	CD141	CD1C	DoubleNeg	pDC	Total number of cells
Batch 1	94	0	94	94	282
Batch 2	0	94	93	96	283

**Table 7:** The variance of each batch in the human dendritic cells’ dataset.

	The variance of CD141	The variance of CD1C	The variance of DoubleNeg	The variance of pDC
Batch 1	1.896093	0	1.900573	1.942468
Batch 2	0	1.862784	1.871015	1.873557

**Table 8:** The ASW score of the batch effects correction methods.

Methods	GIR-Combat	MNN	Combat	Uncorrected	Harmony	Seurat	LIGER
$F1_{\mathrm{ASW}}$ score	0.612005	0.545501	0.548631	0.525663	0.504994	0.499649	0.519674

#### 3.2.3 Mouse haematopoietic stem and progenitor cells datasets

The mouse haematopoietic stem dataset (SMART-seq2) (Nestorowa et al., 2016) profiles 1,656 FACS-sorted hematopoietic stem and progenitor cells, capturing single-cell heterogeneity and pseudotime dynamics of stem-to-progenitor transitions through index sorting and transcriptional analysis. The myeloid progenitor dataset (MARS-seq) (Paul et al., 2015) focuses on bone marrow progenitors, identifying seven distinct lineage-primed subpopulations without mixed states through deep sequencing of thousands of single cells. These two datasets differ in both biological composition and sequencing platform, presenting a complex cross-lineage and cross-platform integration task, which were first jointly used for benchmarking integration methods by Aumüller et al. (2017).

**Figure 12:**
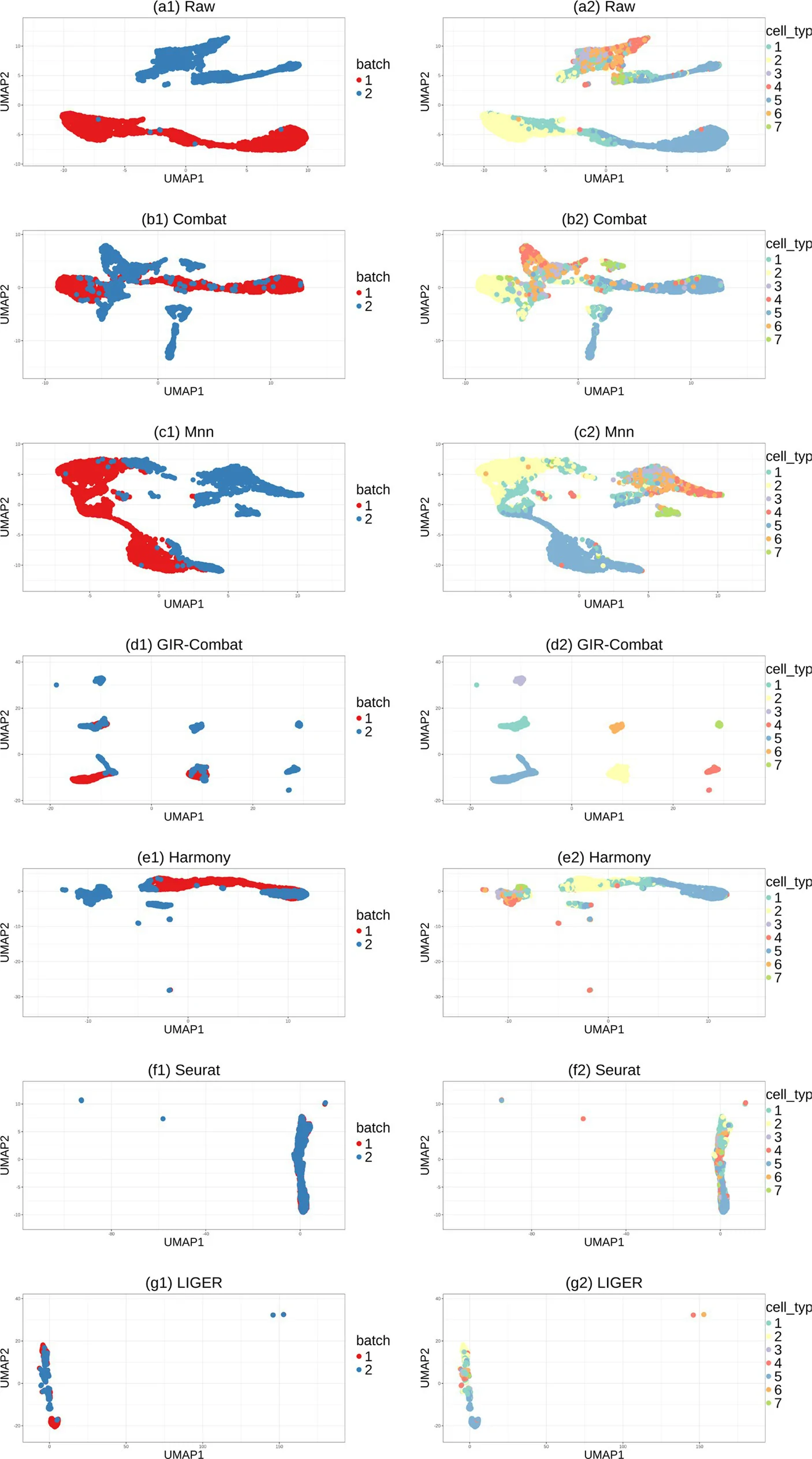
UMAP plots of density visualization of data before and after batch effect correction on Mouse Haematopoietic Stem and Progenitor Cells datasets. **(a)** Batch data, **(b)** batch data corrected by the Combat model, **(c)** batch data corrected by the MNN, **(d)** batch data corrected by the GIR-Combat model, **(e)** batch data corrected by the Harmony, **(f)** batch data corrected by the Seurat, and **(g)** batch data corrected by the LIGER. In each subgraph, for example, a1 represents the UMAP plots of the distribution of batch data by batch, and a2 represents the UMAP plots of the distribution of batch data by traits.

**Figure 13:**
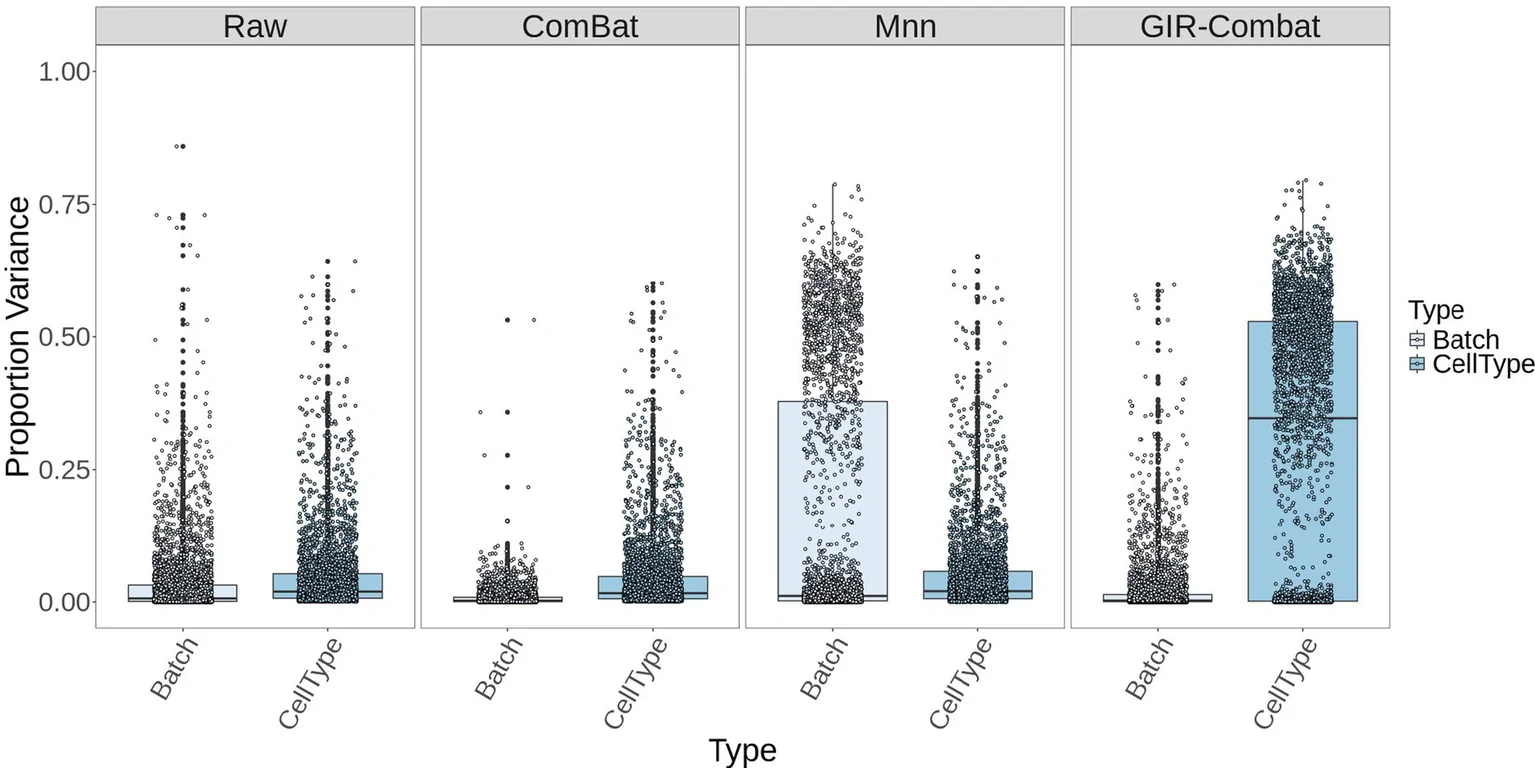
The variance explained plot for the fitted linear mixed model before and after batch effect correction on mouse haematopoietic stem and progenitor cells datasets.

A thorough overview of the dataset is given in Table 9, supporting the characterization that our correction method effectively addresses the imbalances challenge. Table 10 shows the variances of distinct cell types of each batch. Figure 12 presents the UMAP visualizations of the mouse haematopoietic stem and progenitor cells datasets, both prior to and subsequent to the application of batch effect correction. Figure 13 portrays the composition of variance elucidated for this dataset. Meanwhile, Table 11 gives the $F1_{\mathrm{ASW}}$ values for the datasets before and after the implementation of batch effect correction, indicating superior balance between batch effect removal and structure preservation from our approach.

**Table 9:** The cell numbers contained in each batch.

	CMP	GMP	LMPP	LTHSC	MEP	MPP	Unsorted	Total number of cells
Batch 1	480	1,154	0	0	1,095	0	0	2,729
Batch 2	328	123	280	323	362	368	136	1920

**Table 10:** The variance of each batch in the mouse haematopoietic stem and progenitor cells dataset.

	CMP	GMP	LMPP	LTHSC	MEP	MPP	Unsorted
Batch 1	0.476840	0.453961	0	0	0.466235	0	0
Batch 2	0.431503	0.420003	0.428240	0.436396	0.432009	0.433241	0.311936

**Table 11:** The ASW score of the batch effects correction methods.

Methods	GIR-Combat	MNN	Combat	Uncorrected	Harmony	Seurat	LIGER
$F1_{\mathrm{ASW}}$ score	0.586062	0.475073	0.510157	0.427531	0.481006	0.489534	0.429957

#### 3.2.4 Mouse Cell Atlas datasets

The Microwell-seq Mouse Cell Atlas (Han et al., 2018) provides a comprehensive reference using an innovative high-throughput, low-cost scRNA-seq platform, revealing cellular hierarchies across multiple tissues with particular emphasis on previously under-characterized cell types. The Tabula Muris Consortium (SMART-seq2) (Tabula Muris Consortium, 2018) provides a systematic transcriptomic characterization of 20 organs from 3-month-old mice through coordinated large-scale scRNA-seq, creating a standardized baseline for murine cellular diversity. Together, these complementary resources enable systematic exploration in the scenario which profiles identical cell types across batches sequenced using different technologies.

**Figure 14:**
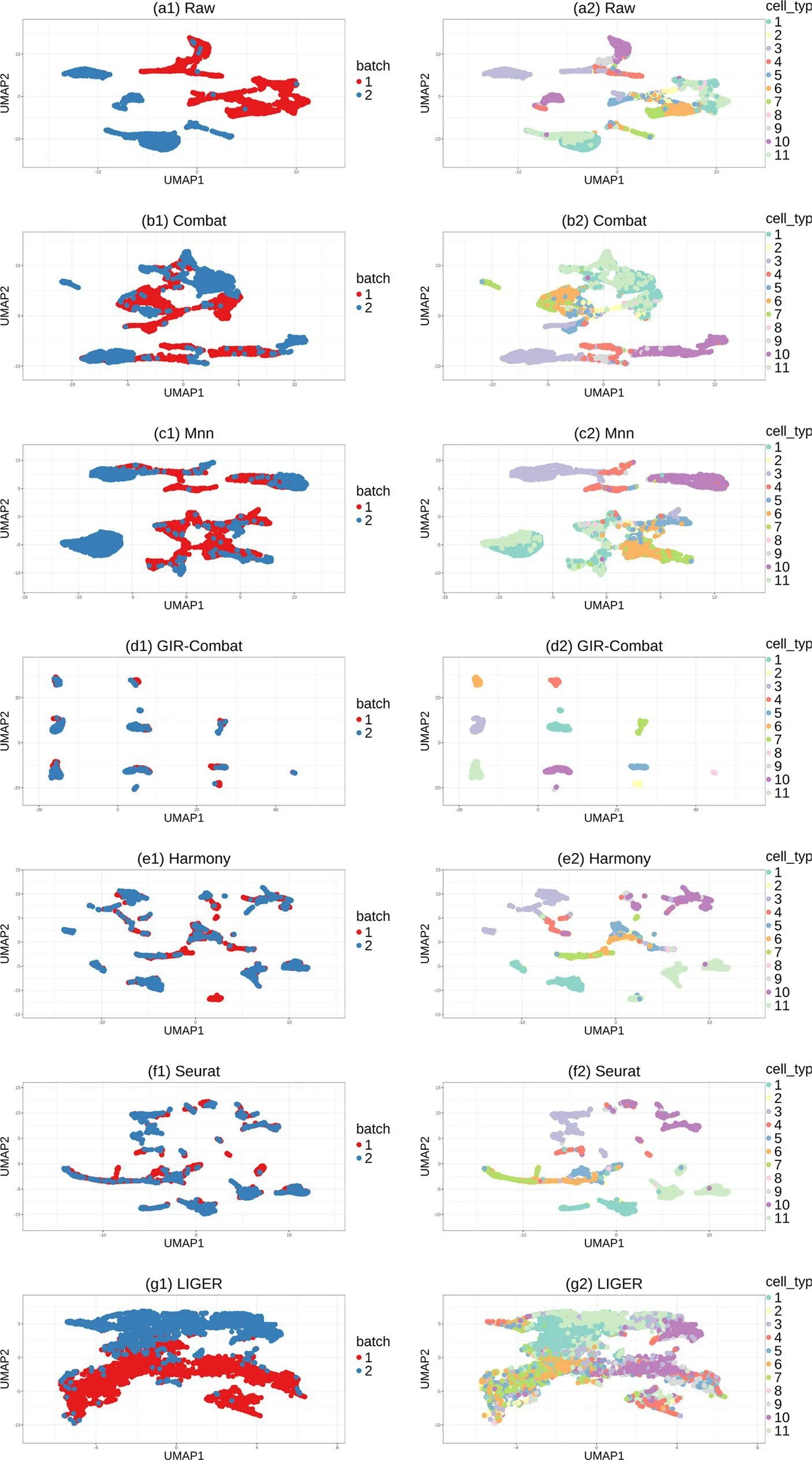
UMAP plots of density visualization of data before and after batch effect correction on Mouse Cell Atlas datasets. **(a)** Batch data, **(b)** Batch data corrected by the Combat model, **(c)** Batch data corrected by the MNN, **(d)** Batch data corrected by the GIR-Combat model, **(e)** Batch data corrected by the Harmony, **(f)** Batch data corrected by the Seurat and **(g)** Batch data corrected by the LIGER. In each sub-graph, for example, a1 represents the UMAP plots of the distribution of batch data by batch, and a2 represents the UMAP plots of the distribution of batch data by traits.

**Figure 15:**
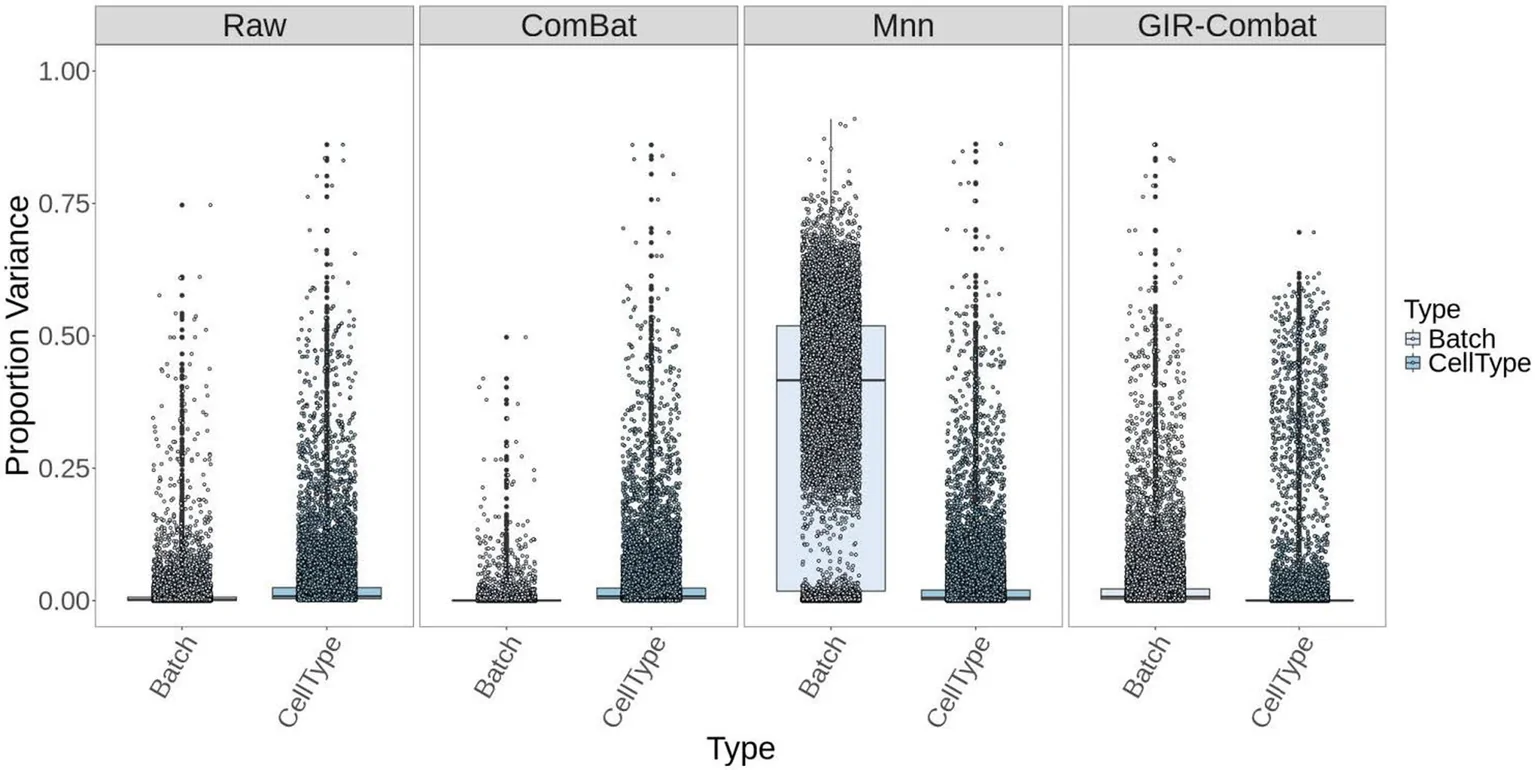
The variance explained plot for the fitted linear mixed model before and after batch effect correction on mouse cell atlas datasets.

Table 12 provides a comprehensive summary of the dataset. Table 13 shows the variances of distinct cell types of each batch. Figure 14 presents the UMAP visualizations of the mouse cell atlas datasets, both prior to and subsequent to the application of batch effect correction. Figure 15 portrays the composition of variance elucidated for this dataset. Meanwhile, Table 14 presents the $F1_{\mathrm{ASW}}$ values for the datasets before and after the implementation of batch effect correction, highlighting the strength of our virtual reference design.

**Table 12:** The cell numbers contained in each batch.

	B-cell	Dendritic	Endothelial	Epithelial	Macrophage	Monocyte	Neutrophil	NK	Smooth-muscle	Stromal	T-cell	Total number of cells
Batch 1	325	202	432	355	425	386	311	36	97	701	969	4,239
Batch 2	781	18	702	35	44	94	143	49	66	331	452	2,715

**Table 13:** The variance of each batch in the human dendritic cells’ dataset.

	B-cell	Dendritic	Endothelial	Epithelial	Macrophage	Monocyte	Neutrophil	NK	Smooth-muscle	Stromal	T-cell
Batch 1	0.239178	0.233303	0.240226	0.236887	0.235815	0.233167	0.215846	0.239843	0.239125	0.234459	0.240642
Batch 2	0.221231	0.221634	0.220263	0.202833	0.202736	0.198222	0.184689	0.214637	0.215575	0.201285	0.223685

**Table 14:** The ASW score of the batch effects correction methods.

Methods	GIR-Combat	MNN	Combat	Uncorrected	Harmony	Seurat	LIGER
$F1_{\mathrm{ASW}}$ score	0.631202	0.515862	0.537657	0.480315	0.504502	0.508653	0.413141

#### 3.2.5 Human peripheral blood mononuclear cell datasets

Human peripheral blood mononuclear cell datasets include scRNA-seq data of human PBMCs (Zheng et al., 2017) generated using two distinct 10x Genomics protocols, 3′ and 5′ mRNA capture, introducing protocol-driven batch effects. The 3′ data was processed using Chromium Single Cell 3′ v2 chemistry, while the 5′ data was generated with Chromium Single Cell 5′ paired-end chemistry.

**Figure 16:**
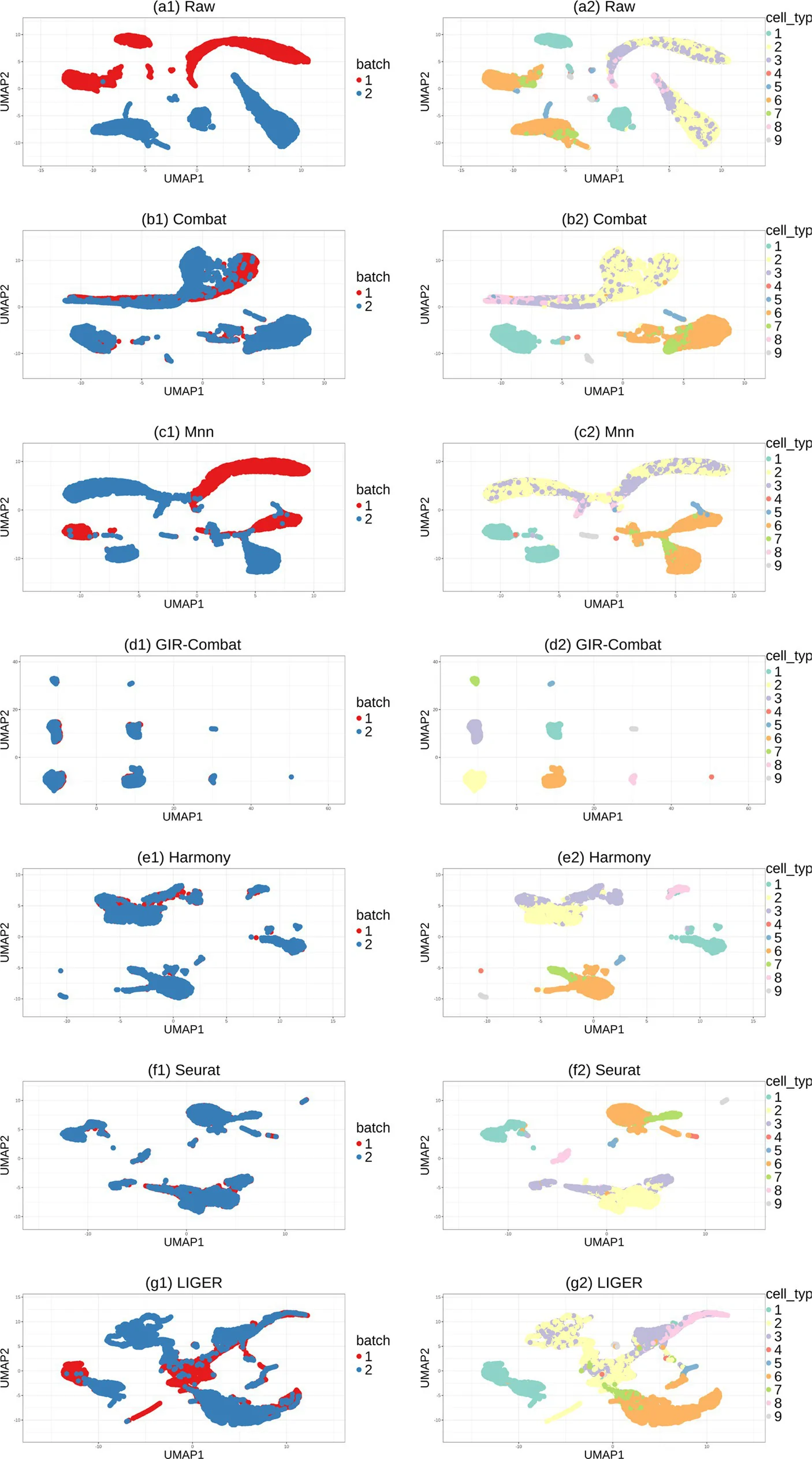
UMAP plots of density visualization of data before and after batch effect correction on Human Peripheral Blood Mononuclear Cell datasets. **(a)** Batch data, **(b)** Batch data corrected by the Combat model, **(c)** Batch data corrected by the MNN, **(d)** Batch data corrected by the GIR-Combat model, **(e)** Batch data corrected by the Harmony, **(f)** Batch data corrected by the Seurat, and **(g)** Batch data corrected by the LIGER. In each sub-graph, for example, a1 represents the UMAP plots of the distribution of batch data by batch, and a2 represents the UMAP plots of the distribution of batch data by traits.

**Figure 17:**
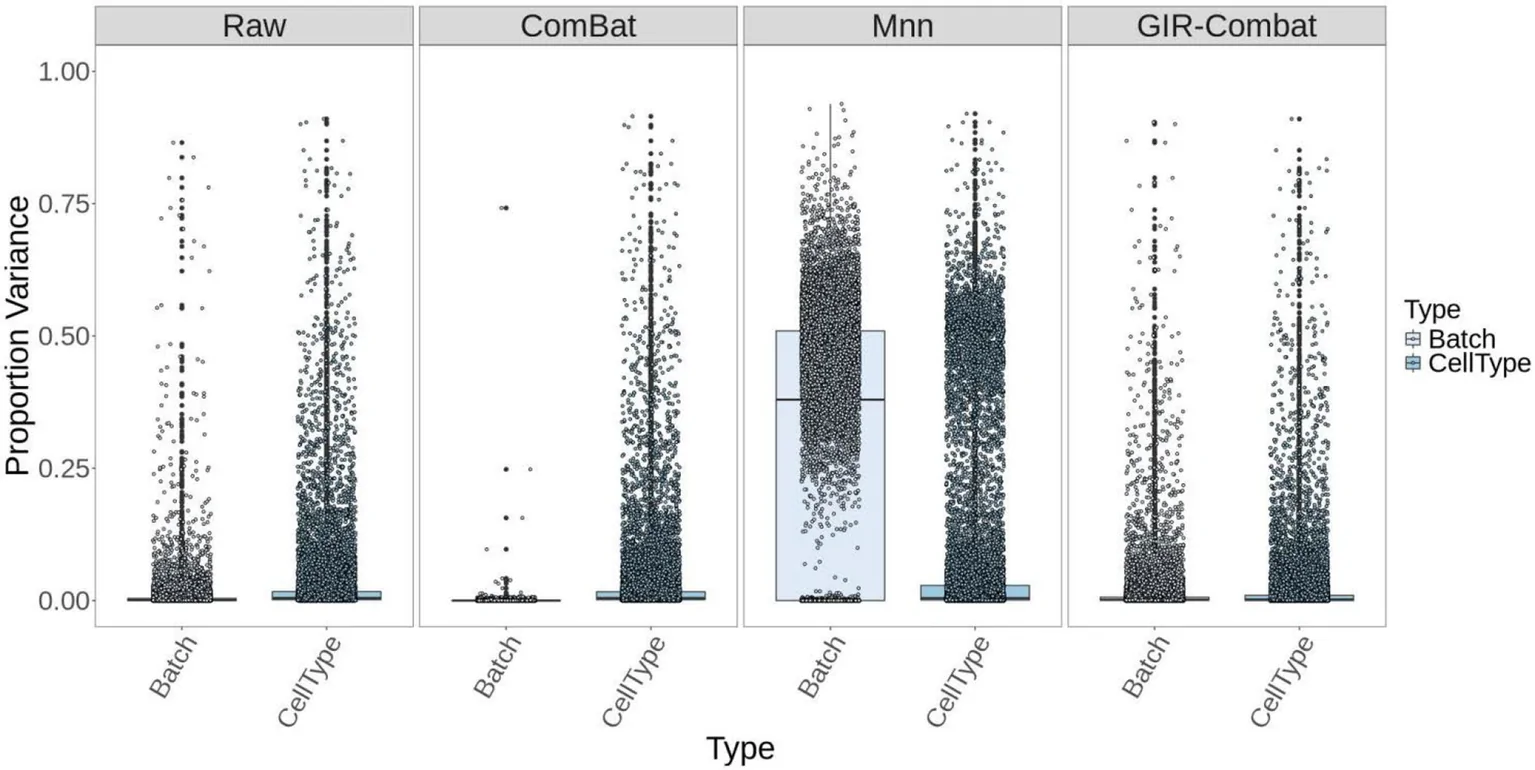
The variance explained plot for the fitted linear mixed model before and after batch effect correction on human peripheral blood mononuclear cell datasets.

Table 15 summarizes the dataset composition, demonstrating that our correction method effectively addresses the protocol-driven batch effects. Table 16 presents the variances of different cell types for each batch. Figure 16 provides the UMAP visualizations of the human peripheral blood mononuclear cell datasets, both prior to and after the batch effect correction. Figure 17 illustrates the variance composition for this dataset. Meanwhile, Table 17 shows the $F1_{\mathrm{ASW}}$ values for the datasets before and after the application of batch effect correction, demonstrating strong structural integrity after correction.

**Table 15:** The cell numbers contained in each batch.

	B cell	CD 4 T cell	CD 8 T cell	Hematopoietic stem cell	Megakaryocyte	Monocyte_CD14	Monocyte_FCGR3A	NK cell	Plasmacytoid dendritic cell	Total number of cells
Batch 1	1,199	2,267	2076	17	49	1914	206	303	67	8,098
Batch 2	1,172	2,183	1,066	7	57	2,176	355	290	72	7,378

**Table 16:** The variance of each batch in the human dendritic cells’ dataset.

	B cell	CD 4 T cell	CD 8 T cell	Hematopoietic stem cell	Megakaryocyte	Monocyte_CD14	Monocyte_FCGR3A	NK cell	Plasmacytoid dendritic cell
Batch 1	0.335910	0.330152	0.333036	0.319562	0.338318	0.339098	0.334561	0.348114	0.336160
Batch 2	0.339336	0.336326	0.339038	0.313596	0.345189	0.346856	0.337313	0.350388	0.337475

**Table 17:** The ASW score of the batch effects correction methods.

Methods	GIR-Combat	MNN	Combat	Uncorrected	Harmony	Seurat	LIGER
$F1_{\mathrm{ASW}}$ score	0.644322	0.520407	0.546003	0.488768	0.522252	0.540949	0.497381

## 4 Discussion

Batch effect correction is crucial in omics data integration analysis, particularly when substantial technical heterogeneity is superimposed on complex biological variability. GIR-Combat addresses a fundamental limitation of conventional Combat by eliminating the need to choose a single reference batch. Instead, GIR-Combat constructs a global-information virtual reference batch derived from MNNs across multiple batches. This approach provides a more biologically relevant baseline for batch correction, ensuring that corrections are guided by shared biological information rather than a predefined batch assignment. Accordingly, the virtual reference should be interpreted as an estimator of shared cross-batch reference structure rather than as a strict probabilistic estimate of a fully latent common distribution.

A key advantage of GIR-Combat is its ability to preserve biological variance while effectively removing batch effects. By integrating MNNs across batches, the method ensures that corrections are applied only where biologically meaningful correspondences exist, thereby minimizing the risks of overcorrection where distinct cell types or states become indistinguishable, and under-correction where batch effects persist. These benefits are especially apparent in high-variance and imbalanced datasets, where conventional methods often fail to maintain biological diversity while removing batch-associated variability.

We validated GIR-Combat on both simulated and real datasets. Compared with commonly used methods, GIR-Combat demonstrates superior performance in effectively handling batch effects while maintaining biologically meaningful separation between cell types or states. These results reinforce the robustness of our virtual reference design, which avoids the need for explicit batch anchoring and adapts to varying degrees of population overlap. This property is especially important when different batches contain complementary rather than redundant information, because under such circumstances a single-batch reference may fail to capture the full structure of the data. The quantitative and visual results together support that GIR-Combat provides a stable balance between batch removal and biological preservation. Combat, while effective in many cases, is highly dependent on the choice of reference batch, which introduces significant biases. MNN-based correction, as originally proposed by Haghverdi et al., is effective in aligning cell states locally but may struggle when batch effects introduce large-scale distortions. It is important to note that in all datasets, the MNN method was applied using its publicly released implementation without dataset-specific tuning. This may partly explain the relatively high residual batch-associated variance observed in MNN’s outputs. Harmony performs well under certain conditions but shows limitations in datasets with pronounced imbalance across batches. Seurat and LIGER, while powerful for data integration, do not always effectively remove batch effects when variance is high and data is imbalanced.

Another important point is the design purpose of the evaluation framework. Rather than relying on a single biological application, we assessed GIR-Combat using controlled simulations together with public datasets spanning distinct correction scenarios. These included high-variance bulk expression data, imbalanced single-cell datasets, partial-overlap settings, cross-platform integrations, and protocol-specific batch effects. Taken together, these datasets were selected to explore conditions under which reference-batch specification becomes especially unstable, namely high variance, compositional imbalance, heterogeneous batch structure, and incomplete cross-batch correspondence. This benchmarking strategy was designed to establish the methodological basis of GIR-Combat across a range of representative correction scenarios, rather than to optimize the framework for one particular biological setting.

Moreover, GIR-Combat is not restricted to a single data modality. As formulated here, the framework operates on sample-by-feature data matrices and is therefore applicable to a broader range of high-variance omics contexts in which biologically comparable groups are shared across batches or can be approximated through clustering. This broader applicability is consistent with the increasing need for robust integration across transcriptomic, proteomic, and other omics data types. In the present study, we evaluated GIR-Combat on representative bulk-expression and single-cell transcriptomic benchmarks. Evaluating the framework in additional omics contexts would be an important direction for future work.

Meanwhile, GIR-Combat presents certain limitations. The method is most effective when batches share at least partially overlapping biological groups, as which provides sufficient anchor pairs for reliable correction. When overlap is limited or absent, MNN-based matching may be insufficient, leading to suboptimal correction. Clustering-based pseudo-label strategies may be required. Hence, its performance depends on the robustness of the clustering step, which may propagate errors into downstream correction. Additionally, the computation of MNN pairs across batches and iterative reference construction may introduce result in increased computational overhead relative to simpler batch correction approaches. Lastly, like other methods, GIR-Combat should not be regarded as universally optimal across all data structures. It is expected to be most effective in the high-variance and compositionally imbalanced cases for which it was designed.

These considerations are particularly relevant in applications where integrated omics data serve as the basis for inference in interaction-rich biological systems. In studies of microbial symbiosis, host–microbe interactions, and ecosystem robustness, downstream interpretation often depends on whether integration preserves biologically meaningful cross-sample structure rather than merely minimizing technical variation. From this perspective, the value of GIR-Combat lies not in directly inferring interaction mechanisms, but in providing a more reliable integrated representation on which such inference can be built. Future work will be to extend the evaluation of GIR-Combat to symbiosis-oriented and host-associated omics datasets, where reliable cross-batch integration directly affects the interpretation of interaction-rich biological systems. Such analyses would provide a more direct assessment of the utility of GIR-Combat in these applications.

In summary, our results demonstrate that GIR-Combat outperforms traditional methods in high-variance and imbalanced settings, effectively achieving batch correction while retaining biologically meaningful differences. By constructing a virtual reference batch and implementing iterative corrections, GIR-Combat reduces the arbitrariness of reference batch selection, systematically removes batch effects, and preserves critical biological variation. These findings highlight the potential of GIR-Combat as a reliable and generalizable batch correction approach, especially for datasets with high technical noise and complex batch structures.

## 5 Conclusion

In this study, we presented GIR-Combat, an enhanced reference-based batch correction framework for high-variance omics data. GIR-Combat employs a global-information virtual reference batches, constructed through pairwise nearest neighbor comparisons, to provide more objective and robust batch correction strategy compared to traditional methods. It transforms reference specification from an externally imposed choice into a data-driven component of the correction process.

Across simulated and public omics datasets, GIR-Combat consistently improved batch correction performance relative to existing methods. These results demonstrate its effectiveness in addressing batch effects, thereby enhancing the comparability and reliability of high-variance omics datasets. Specifically, GIR-Combat improved calibration robustness and reduced subjectivity in reference-batch selection, thereby addressing a key limitation of existing reference-based methods. Our findings establish GIR-Combat as a robust, scalable, and generalizable framework for challenging multi-batch integration tasks. By reducing the arbitrariness of reference specification while maintaining critical biological variation, GIR-Combat provides a practical and methodologically grounded solution for challenging batch correction tasks and offers a promising basis for future applications in interaction-rich biological systems.

## Data Availability

The source code is available at: https://github.com/BioStatAnalysis1/GIR-Combat for academic usages only. The original contributions presented in the study are included, further inquiries can be directed to the corresponding author.
