# Supplementary material for: A novel batch effect correction framework for robust integration of high-variance data via a global-information virtual reference batch
Source: Front Microbiol. 2026 Aug 12;17:1877381. doi: 10.3389/fmicb.2026.1877381 (PMC13506711; doi:10.3389/fmicb.2026.1877381)
Supplement: Supplementary file 1 [file Data_Sheet_1.DOCX]

**Supporting information for**

A novel batch effect correction framework for robust integration of high-variance data via a global-information virtual reference batch

Yuqian Liu^1,2, †^, Lan Du^1, 2, †^, Jingxuan Jiang^1^, Xin Tong^1^, Junfei Xu^1^, Jiayin Wang^1,2^, Xin Lai^1,2,^*

^1^School of Computer Science and Technology, Faculty of Electronics and Information Engineering, Xi’an Jiaotong University, Xi’an, Shaanxi, China

^2^Shaanxi Engineering Research Center of Medical and Health Big Data, Xi’an Jiaotong University, Xi’an, Shaanxi, China

^†^These authors have contributed equally to this work and share first authorship

*** Correspondence:**
Xin Lai: laixin@xjtu.edu.cn

**Appendix A. Using Annoy algorithm to search for mutual nearest neighbors**

The Annoy algorithm necessitates the construction of an index prior to identifying the mutual nearest neighbors of samples. Recursively partition the space containing samples to generate multiple binary search trees, with the number of binary search trees determined by the user's specifications. The nodes of the binary search trees represent subdivided subspaces, with leaf nodes storing the samples. Each subdivided subspace is determined by a random hyperplane, defined by a randomly selected unit vector, which projects the samples onto the randomly selected vector, thereby partitioning the space. For each sample vector $\vec{E}^{x}$, the dot product with the randomly selected unit vector $\vec{e}$ is computed; if the dot product is positive, the sample is assigned to the right child node, and if negative, to the left child node. Following the construction of the index, the algorithm commences the search for mutual nearest neighbor sample pairs. The formula for calculating the dot product is shown in Equation (10). In the formula: $\theta$ denotes the angle between the sample vector$\vec{x}$ and a randomly chosen unit vector $\vec{e}$, expressed as their dot product.

$$\begin{aligned} \vec{E}^{x}\cdot\vec{e}=|\vec{E}^{x}||\vec{e}|cos\theta=|\vec{E}^{x}|cos\theta\#\left( 10 \right) \end{aligned}$$

Searching for mutual nearest neighbor sample pairs starts with a sample nearest neighbor lookup. First, establish an empty list for the nearest neighbors, then traverse each binary search tree commencing from the root. At each node within the tree, ascertain the side of the tree split to which the present query sample belongs. The search process is steered by a priority queue Q, which stores the root node of each binary tree. This queue monitors the current samples to be queried. The queue is organized according to the distance from the sample to the defining hyperplane of the partition, with closer distances being more likely to be added to the queue Q. If the priority queue Q is not empty, the candidate node with the shortest distance is dequeued. If the current candidate node constitutes a leaf node, the candidate sample is queried, and the distance between the query sample and the candidate sample is computed. The process of finding the nearest neighbor for a single sample is shown in **Fig. S1**.

**Fig. S1.** Flowchart for finding the nearest neighbour of a single sample
